# Surface Exclusion Revisited: Function Related to Differential Expression of the Surface Exclusion System of *Bacillus subtilis* Plasmid pLS20

**DOI:** 10.3389/fmicb.2019.01502

**Published:** 2019-07-10

**Authors:** César Gago-Córdoba, Jorge Val-Calvo, Andrés Miguel-Arribas, Ester Serrano, Praveen K. Singh, David Abia, Ling Juan Wu, Wilfried J. J. Meijer

**Affiliations:** ^1^Laboratory 402, Centro de Biología Molecular “Severo Ochoa” (CSIC-UAM), Department of Virology and Microbiology, Autonomous University of Madrid, Madrid, Spain; ^2^Centre for Bacterial Cell Biology, Institute for Cell and Molecular Biosciences, Newcastle University, Newcastle upon Tyne, United Kingdom

**Keywords:** surface exclusion, antibiotic resistance, Firmicutes, horizontal gene transfer, conjugation, Gram-positive bacteria, gene expression, surface protein

## Abstract

During conjugation a genetic element is transferred from a bacterial donor to a recipient cell via a connecting channel. It is the major route responsible for the spread of antibiotic resistance. Conjugative elements can contain exclusion system(s) that inhibit its transfer to a cell already harboring the element. Our limited knowledge on exclusion systems is mainly based on plasmids of Gram-negative bacteria. Here we studied the conjugative plasmid pLS20 of the Gram-positive *Bacillus subtilis*. We demonstrate that pLS20 contains an exclusion system and identified the single gene responsible for exclusion, named *ses_pLS20_*, which is embedded in the conjugation operon. Ses_pLS20_ is the founding member of a novel family of surface exclusion proteins encoded by conjugative elements of Gram-positive origin. We show that the extent of surface exclusion correlates with the level of *ses_pLS20_* expression, and that *ses_pLS20_* is expressed at basal low-levels in all donor cells but becomes highly expressed in conjugating cells. Accordingly, the transfer of pLS20 from a conjugation-primed donor cell to an un-primed or conjugation-primed donor is inhibited moderately and very efficiently, respectively. The consequences of this differential regulation, which appears to be a conserved feature of surface exclusion systems of Gram-positive and Gram-negative origin, are discussed.

## Introduction

Horizontal gene transfer (HGT) refers to the transfer of DNA regions from one cell to another which happens at large scale in bacteria (for review see, Ochman et al., 2000; Frost et al., 2005; Thomas and Nielsen, 2005). HGT plays a major role in the evolution of bacteria, but unfortunately is also responsible for the emergence and dissemination of antibiotic resistance (Mazel and Davies, 1999). The World Health Organization has highlighted the problem with antibiotic resistance and warned that the problem is getting worse. Of the various HGT mechanisms, conjugation is the principal route that is responsible for the emergence and dissemination of antibiotic resistance (Mazel and Davies, 1999). Conjugation is the process by which a conjugative element is transferred from a donor to a recipient cell via a connecting channel. Conjugative elements can be embedded in the bacterial genome (named integrative and conjugative elements; ICE) or present on plasmids, which are named conjugative plasmids. Most conjugation studies are based on a limited number of plasmids of Gram-negative (G−) origin. Antibiotic resistance is a problem though in both G− and Gram-positive (G+) bacteria, particularly in bacteria belonging to the phylum of Firmicutes (Sommer et al., 2009). We have been studying the native conjugative plasmid pLS20 from the G+ Firmicutes *Bacillus subtilis*. *Bacillus* strains form part of the microbiome of humans and animals and Bacilli are widely used as probiotics in animals. pLS20cat, a derivative of pLS20 containing a chloramphenicol-resistance gene, contains a large conjugation operon encompassing genes *28*–*74* (according to our gene annotation) that is under the control of the strong promoter P*_c_*. Expression of the conjugation genes is regulated by genes *25*–*27*. Gene *27* encodes the master regulator of conjugation, Rco_pLS20_, which binds to the operator sites located near the divergently oriented promoters P*_c_* and P*_r_*, the latter driving expression of *rco_pLS20_*. This binding results in repression of P*_c_* promoter and activation of the P*_r_* promoter thereby keeping conjugation in the default “OFF” state (Singh et al., 2013; Ramachandran et al., 2014). Gene *25* encodes an anti-repressor, Rap_pLS20_, whose activity is regulated by the signaling molecule Phr^∗^_pLS20_, that is encoded by gene *26* (Singh et al., 2013; Ramachandran et al., 2014). In this study we addressed the question whether pLS20cat contains an exclusion system and what role(s) it plays in conjugation.

Exclusion systems inhibit the transfer of a conjugative element to a cell already harboring the element. They were first described more than half a century ago (Lederberg et al., 1952). Despite many studies, their mechanisms of action remain poorly understood. *Escherichia coli* plasmid F and related plasmids have been studied in most depth. The F plasmid contains two genes, *traS* and *traT*, each encoding for a protein responsible for a distinct type of exclusion, named entry exclusion (EE; *traS*) and surface exclusion (SE; *traT*). Other conjugative plasmids of G− origin contain either a surface or an EE system, or both. EE systems appear to be more effective than SE systems (Garcillan-Barcia and De la Cruz, 2008). The TraS EE system involves interaction of two inner membrane proteins present on the two mating cells: TraS on the recipient cell and TraG on the conjugation-primed donor cell (Achtman et al., 1979; Anthony et al., 1999; Audette et al., 2007), which causes abortion of the conjugation process after stable mating pairs have been formed (Ou, 1975; Hartskeerl and Hoekstra, 1984). The *traT* SE gene, on the other hand, encodes an outer membrane protein and its presence apparently reduces the ability of the cell to form a stable mating aggregate (Achtman et al., 1977).

Far less is known about exclusion systems present on conjugative plasmids of G+ origin. Here we show that pLS20cat contains an exclusion system. We identified gene *29* as the only gene responsible for exclusion, and name it *ses_pLS20_*. We show that Ses_pLS20_ is a surface-located protein and that it is the prototype of a new family of SE proteins encoded by multiple conjugative elements present in G+ bacteria, including pathogens. The principal function of exclusion systems is generally believed to prevent redundant transfer of conjugative elements. Interestingly, we found that *ses_pLS20_*, which is located within the conjugation operon, is not only controlled by the strong conjugation P*_c_* promoter, but that it is also expressed from a weak constitutive promoter, P*_*29*_*. Consequently, *ses_pLS20_* is weakly expressed in all donor cells and strongly expressed in those donor cells having switched on the conjugation process. We also show that the level of exclusion correlates with the level of *ses_pLS20_* expression. This means that the main function of the exclusion system is not to prevent redundant transfer *per se* but that this is restricted to the minor population of conjugation-primed donor cells. Probably this type of regulation applies to many or perhaps all other SE systems known and hence our findings shed a new light on the role of the exclusion systems in general.

## Materials and Methods

### Bacterial Strains, Plasmids, Media and Oligonucleotides

*Bacillus subtilis* and *Escherichia coli* strains were grown in Luria-Bertani (LB) liquid medium or on 1.5% LB agar plates. When appropriate, media were supplemented with the following antibiotics: ampicillin (100 μg/ml), spectinomycin (100 μg/ml), chloramphenicol (5 μg/ml), erythromycin (1 and 150 μg/ml in *B. subtilis* and *E. coli*, respectively), and kanamycin (10 and 30 μg/ml in *B. subtilis* and *E. coli*, respectively). All *B. subtilis* strains used were isogenic with *B. subtilis* strain 168 (see Supplementary Table S1). Plasmids and oligonucleotides (Isogen Life Science, Netherlands) used are listed in Supplementary Tables S2, S3, respectively.

### Transformation

*Escherichia coli* cells were transformed using standard methods (Sambrook et al., 1989). Generation of competent *B. subtilis* cells and transformation were done as before (Bron et al., 1989).

### Construction of Plasmids and Strains

All cloned PCR fragments were checked for their correctness by sequence analysis. Total DNA extracted from pLS20cat harboring strain PKS11 was used as template to amplify pLS20cat regions by PCR.

Plasmid pLS20 was labeled with a spectinomycin resistance gene as follows. Plasmid pCm::Sp, designed to replace a chloramphenicol resistance (*cat*) gene by a spectinomycin resistance (*spec*) gene (Steinmetz and Richter, 1994), was used to transform competent *B. subtilis* cells of strain PKS56, which harbors pLS20cat. Spectinomycin-resistant transformants were checked for sensitivity to chloramphenicol. The total DNAs isolated from several Spec^R^/Cm^S^ clones were used as templates in PCR reactions to confirm replacement of the *cat* by the *spec* gene. Next, the total DNA of a representative clone, named PKS76, was used to transform competent cells of *B. subtilis* strain 168. The presence of pLS20spec in a spectinomycin-resistant transformant was demonstrated by PCR and conjugation experiments. The resulting strain was named PKS91.

The promoter strength screening vector pKSsfGFP is a derivative of pJS104, which is an *amyE* integration vector containing a *gfp* reporter gene that has been modified in multiple ways to enhance its expression and stability: it has an extension of eight codons of the *B. subtilis comGA* gene at the N-terminus (Veening et al., 2004); it has a changed codon bias compromised to obtain optimum expression in both *E. coli* and *B. subtilis*; it contains the *mut*3 *gfp* mutations (S2R, S65G, S72A) (Andersen et al., 1998) combined with the superfolder mutations (S30R, Y39N, F64L, G65T, F99S, N105T, Y145F, M153T, V163A, I171V, A206V) (Pedelacq et al., 2006), and is monomeric in solution (Pedelacq et al., 2006). For simplicity we refer to this gene as *gfp*. The *gfp* gene in pJS104 is preceded by a 124 bp *Eco*RI fragment containing the constitutive promoter of bacteriophage SPO1 gene *26*, and two unique restriction sites (*Bam*HI, *Hin*dIII). The following strategy was used to replace the promoter region by a 25 bp region including additional unique restriction sites (*Eco*RI, *Nhe*I and *Spe*I). A 328 bp DNA fragment overlapping with the N-terminus of the *gfp* gene was amplified by PCR with primer set [oPKS45-oSeqpKSGFP_Dn] and using pJS104 as template DNA. This fragment was digested with *Eco*RI and *Kpn*I and the 224 bp was purified and cloned into pJS104 digested with the same enzymes. The resulting pKSsfGFP vector contains a promoter less *gfp* gene that is preceded by the unique restriction sites *Bam*HI, *Hin*dIII, *Eco*RI, *Nhe*I, and *Spe*I.

The pKSsfGFP vector was used to construct *B. subtilis* strains containing a cassette at the chromosomal *amyE* containing either a promoter-less *gfp* fusion or *gfp* fused to promoter P*_c_* or P*_*29*_*. In the case of the P*_*29*_* promoter, the 358 bp region upstream of pLS20cat gene *29* was amplified with primer set [29_UpHindIII - 29DnHIII]. This PCR fragment was digested with *Hin*dIII and cloned in the *Hin*dIII-linearized vector pKSsfGFP. Colony PCR using primer set [oSeqpJS104_Dn – oSeqpKSGFP_Dn] was used to identify pKSsfGFP derivatives containing the insert, and sequencing of positive clones was performed to confirm the absence of mutations and to select the vector having the insert cloned in the desired orientation. The resulting plasmid was named pCG1 (P*_*29*_*-*gfp*). In the case of the P*_c_* promoter, the 583 bp region containing promoter P*_c_* (Ramachandran et al., 2014) was amplified using primer set [Prom28UP_Hind - Prom28Dn_Bam]. After purification, the 3′-ends of the fragment were extended with an ATP nucleotide by incubating the fragment with *Taq* DNA polymerase in the presence of dATP at 73°C for 20 min. Next, the purified fragment was cloned into the TA cloning vector pTZ57R/T (Thermo Fisher Scientific, United States). The absence of mutations in the plasmid isolated from a white transformant growing on LB agar plate supplemented with ampicillin, isopropyl β-D-1-thiogalactopyranoside (IPTG, 1 mM) and 5-bromo-4-chloro-indolyl-β-D-galactopyranoside (Xgal; 40 μg/ml) was confirmed by sequencing. The resulting plasmid was named pTAP28Hind_B. Next, the *Hin*dIII fragment containing promoter P*_c_* of pTAP28Hind_B was cloned into the *Hin*dIII linearized vector pKSsGFP. Colony PCR using primer set [oSeqpJS104_Dn – oSeqpKSGFP_Dn] was used to identify the pKSsGFP derivative containing the insert, and sequencing of positive clones was performed to select the vector having the insert cloned in the desired orientation. The resulting vector was named pAND2A (P*_c_*-*gfp*). Plasmids pKSsfGFP, pCG1 and pAND2A were isolated from the corresponding *E. coli* strains and used to transform competent *B. subtilis* 168 cells. Transformants were selected on LB plates containing spectinomycin. Double-crossover integration into the chromosome was checked by the loss of amylase activity.

The *gfp* reporter gene and the pLS20cat genes *29* and/or *30* were cloned behind the IPTG-inducible P*_spank_* or P*_hyspank_* promoter in the *B. subtilis amyE* integration vector pDR110 (P*_spank_* promoter) or pDR111 (P*_hyspank_* promoter) using a similar strategy. The genes were amplified by PCR using appropriate primer sets (oCG11-oCG12, oEST13-oEST17, oEST18-oEST14, and oEST13-oEST14 for cloning of gene(s) *gfp*, *29*, *30*, and [*29*–*30*], respectively). Plasmid pKSsfGFP was used as template to amplify the *gfp* gene. After purification, the PCR fragments were digested with [*Hin*dIII and *Sal*I] (in the case of the *gfp* containing fragment), or with [*Sal*I and *Sph*I] (in the case of the other fragments) and then ligated with the vector pDR110 or pDR111 cut with the same enzymes. Plasmid DNA of the constructed vectors pCG35 (P*_spank_*-*gfp*), pCG36 (P*_hyspank_*-*gfp*), pCG2 (P*_spank_*-*29*), pCG3 (P*_spank_*-*30*), pEST19 (P*_spank_*-*29-30*), and pCG106 (P*_hyspank_*-*29*) was isolated from *E. coli* cells and used to transform competent *B. subtilis* 168 cells. Transformants were selected on LB plates containing spectinomycin. Double-crossover integration into the chromosome was checked by the loss of amylase activity.

Strain CG129, which contains an IPTG-inducible *ses_pLS20_-cMyc* fusion at the *amyE* locus, was constructed as follows. In a first PCR reaction the pLS20cat gene *29* was amplified and extended in frame with a “GGGGS” linker region. For this PCR, pLS20cat was used as template DNA in combination with primer set oEST13-oCG64. The cMyc encoding tag was then added in frame to the linker region in two additional PCR reactions. In the first one, the amplified product of the foregoing PCR was used as template in combination with primer set oEST13-oCG67. In the final PCR, the product of the foregoing PCR was used as template in combination with primer set oEST13-oCG68. The final PCR product was purified, digested with *Sal*I and *Sph*I and used to clone into vector pDR111 digested with the same enzymes. The resulting vector, pCG129, was then used to transform competent *B. subtilis* 168 cells. Spectinomycin-resistant transformants were checked for double cross-over of the cassette at the chromosomal *amyE* locus by loss of amylase activity. Strain CG133 (*amyE*::P*_hyspank_*-*ses_pLS20_-cMyc*, *lacA*::P*_xyl_*-*gfp*) was constructed by transforming competent CG129 cells with plasmid pAX01-sfGFP (see below construction of pAX01-sfGFP). Several erythromycin resistant transformants were screened for double cross-over of the P*_xyl_*-*gfp* cassette at *lacA* by PCR. Thus, total DNAs of transformants were used as templates in PCR reactions with primer sets [opAX1up-opAX1Dn] and [oPAX2Up-oPAX2Dn]. Control strain CG47 (*lacA*::P*_xyl_*-*gfp*) was constructed using the same strategy as for constructing CG133 but using competent cells of wild type strain 168, instead of CG129.

Plasmid pAX01-sfGFP was constructed as follows. The *gfp* gene was amplified by PCR from the pKSsfGFP plasmid with primer set oCG26-oCG27. The resulting PCR fragment was digested with *Bam*HI and cloned into the *Bam*HI site of pAX01. Transformants were checked for the presence of the insert by PCR using primer set pAXseqDn-pAXseqUp, and by screening cells grown in the presence of xylose for fluorescence.

Marker less in-frame deletions of either gene *29* or *30*, or both [*29–30*] on pLS20cat were constructed making use of the pMiniMAD2 vector (Patrick and Kearns, 2008). The method is based on single cross-over integration of a temperature-sensitive replicon via homologous recombination at restrictive temperature followed by permitting replication of the integrated replicon by growth at the permissive temperature to provoke deletion of replicon thereby generating the desired deletion (Arnaud et al., 2004). The pMiniMAD2 vector is based on the *E. coli* vector pUC19, and contains a fragment of the pE194 rolling-circle plasmid encompassing its erythromycin resistance gene and its replication functions. Hence, it can replicate in *B. subtilis* and is selected by erythromycin. However, the pE194 replication gene contains a mutation causing the encoded replication protein to be temperature sensitive. The deletion strategy is based on the following procedure. Regions of ∼600 bp flanking the up- and downstream region to be deleted were amplified and fused by overlapping PCR, and subsequently cloned into the pMiniMAD2 vector. The resulting plasmid was isolated from the RecA-proficient *E. coli* strain JM101 and used to transform freshly prepared competent *B. subtilis* cells containing pLS20cat (strain PKS11). After transformation, cells were plated on LB plates supplemented with erythromycin and incubated overnight at 37°C forcing the pMiniMAD2-derivative to integrate into pLS20cat via a single cross-over event. Several transformants were randomly selected and grown in liquid medium without antibiotic pressure during 14–16 h at 22°C. Under these latter conditions the replicons of both pLS20cat and pE194 are functional creating stress that can be relieved by homologous recombination which results in either the deletion of the entire pMiniMAD2-derivative plasmid or by deletion of the desired region. Thus, after growth at 22°C appropriate dilutions of the cultures were plated on LB plates supplemented with chloramphenicol. After overnight growth at 37°C at least 100 colonies were tooth picked on plates containing erythromycin to select clones that had lost the pE194 replicon including the erythromycin resistance gene. Next, the total DNA of several erythromycin-sensitive colonies was isolated and used as template in PCR reactions to check for deletion of the desired fragment. The following primer sets were used to generate the different in frame deletions. Gene *29*: [Up fragment, D29_1 and oCG28] [down fragment, oCG29 and oCG30]. Gene *30*: [Up fragment, D30_p8 and Connect_C29_C30_P9] [down fragment, D29_30_p6 and D29_30_p7]. Gene *29*-*30*: [Up fragment, D29_1 and connectN29_C30_p5] [down fragment, D29_30_p6 and D29_30_p7]. For each deletion, first the “UP” and “Down” regions were generated by PCR. Next, equal amounts (generally corresponding to 100 ng) of each purified PCR fragment were used for preparing a PCR reaction mixture lacking primers. This mixture was then used to fuse both fragments by performing extension reactions (50 μl) using the following settings [(2 min 94°C); 13 rounds (15 s 94°C; 1 min 55°C; 1.5 min 73°C)]. Next, 2 μl of this elongation reaction was used as template in a subsequent conventional PCR reaction (100 μl) containing the appropriate outer primers using the following settings [(4 min 94°C); 30 rounds (30 s 94°C; 30 s 55°C; 1.5 min 73°C)]. The resulting fused PCR product was purified and digested with *Hin*dIII and *Sal*I and cloned into the pMiniMAD2 vector digested with the same enzymes. The following primer sets were used to check deletion of the desired regions by PCR using as template total DNA of erythromycin-sensitive clones. Gene *29*: oCG14-oCG15; gene *30*: oCG18-oCG19; gene *29-30*: oCG16-oCG17. Consequently, the following in frame deletions were created. Gene *29*: codon eight fused to codon 370; Gene *30*: codon three fused to codon 79; Gene *29–30*: codon three of gene *29* fused to codon 79 of gene *30*.

### *In silico* Analyses

#### Identification of Membrane and/or Surface Proteins

Deduced pLS20cat protein sequences of all ORFs larger than 40 residues were screened online for the presence of transmembrane spanning domains and their transmembrane topology using the hidden Markov model based TMHMM server version 2.0 of the center for biological sequence analyses of the Technical University of Denmark (DTU ^1^) (Sonnhammer et al., 1998; Krogh et al., 2001). The presence of potential signal peptidase cleavage sites 1 were predicted using the SignalP 4.1 server (DTU^2^) using default settings for proteins of Gram-positive bacteria.

#### Identification of Genes Encoding Proteins Showing Significant Similarity to Ses_pLS20_

Ses_pLS20_ and Ses_p576_ were used separately as a query sequence to execute blastp searches^3^ against the NCBI nr protein database (version 2.5.0+, February 2017) (Altschul et al., 1997, 2005; Schaffer et al., 2001). This search resulted in the detection of 46 sequences sharing significant similarity with Ses_pLS20_ and Ses_p576_. The program “USEARCH”^4^ (version 8.0.1517_i86linux32) was then used to identify and remove redundant sequences showing 100% identity (Edgar, 2010), resulting in 30 unique hits.

#### Crossing Ses_pLS20_ Homologs Against Constructed Plasmid Database

Plasmids deposited in the NCBI nr database were retrieved by screening the annotations for the keywords “plasmid” and “circular DNA.” The 10,904 plasmids retrieved at 6 Nov 2016 were used to build a blast database. Next, each Ses_pLS20_ homolog identified was run against the constructed plasmid database using tblastn. A Ses_pLS20_ homolog member was considered to be located on a plasmid if an identity of more than 95% was identified over more than 80% of the entire protein sequence.

### RNA Isolation, RNA Sequencing and Bioinformatic Analyses of RNAseq Data

RNAseq experiments were performed as described before (Singh et al., 2013). In short, total RNA was isolated from late exponentially growing PKS11 (pLS20cat) or PKS14 (*amyE*::P*_spank_*-*rco_pLS20_*, pLS20cat) cells, and possible traces of DNA were removed with a DNase treatment step. Next, after removal of rRNA, 150–250 ng of RNA was used to prepare cDNA libraries using a procedure that preserves the information about the direction of transcription. Bar-coded fragmented samples were titrated, bound at a final concentration of 10 pM to an Ilumina SR-flowcell. Libraries were then run according to the Illumina “sequencing by synthesis” (SBS) technology under a single-read 1x75 protocol. Quality filtering was performed according Illumina specification and fastq files generated. The data sets analyzed were constituted by two *B. subtilis* subsp *subtilis* strain 168 and plasmid pLS20cat samples with totals of 56⋅10^6^ and 48⋅10^6^ single end reads of 50 nt in FASTQ format, respectively. Quality control of the reads, alignments and calculations of expression levels were performed as described before (Singh et al., 2013).

### Conjugation Assays

Conjugation was carried out in liquid medium as described previously (Singh et al., 2013).

### Flow Cytometry

Overnight grown cultures were diluted 100-fold in pre-warmed LB medium. Two ml of the culture was centrifuged (1 min 14,000 *g*) when the OD_600_ was between 0.8 and 1.0. After a washing step (2 ml 0.2 μM filtered 1xPBS), the pellet was resuspended in 1 ml 0.2 μM filtered 1xPBS. Next, cells were directly measured on a FacsCalibur cytometer (Becton Dickinson, United States) equipped with an argon laser (488 nm). The fluorescence of at least 100,000 cells was analyzed using a 530/30 nm band pass filter using arbitrary units (AU). Sample data was collected using CellQuest Pro (Becton Dickinson, United States) software and afterward analyzed using FlowJo 6.4.1 mac (TreeStar, United States) software. *B. subtilis* strain 168 was included in each flow cytometry experiment as negative control. Values showed and represented in graphs, corresponds with Geomean estimated by flow jo.

## Results

### Terminology Used

In most conjugation systems, including pLS20, the conjugation genes are activated only in a subpopulation of cells (Chatterjee et al., 2011; Singh and Meijer, 2014; Rosch and Graumann, 2015). To distinguish different cells in mating experiments we use the following nomenclature. Donor: cell containing pLS20 or a derivative, regardless whether or not it has activated the conjugation genes. Conjugation-primed donor: cell containing pLS20 (or derivative) that has activated the conjugation process. Recipient: cell amenable to receive a plasmid when it mates with a conjugation-primed donor cell. Receptive-donor: cell containing pLS20 (or derivative) that acts as a recipient cell when mating with a conjugation-primed donor cell.

### pLS20 Contains an Exclusion System

The presence of an exclusion system on a conjugative element would result in lower conjugation efficiencies (CEs) for matings between two donor strains as compared to standard matings (i.e., between donor and plasmid-free recipient strains). Using a standard protocol (Singh et al., 2013), the maximum CE of pLS20cat, a pLS20-derivative labeled with a chloramphenicol marker, at the end of the exponential growth phase is in the order of 10^−3^ transconjugants per donor (Singh et al., 2013, see Table 1). To test the CE between two donor strains we constructed pLS20spec, which carries a spectinomycin resistance marker instead of a chloramphenicol marker. Whereas the CE obtained for pLS20spec in matings with plasmid-free recipient cells was very similar to that obtained with pLS20cat, the CE using two donor strains was about 10-fold lower (see Table 1). This strongly indicated that pLS20 contains an exclusion system.

To facilitate the comparison of the extent of exclusion, we index the exclusion level as done for other exclusion systems (Marrero and Waldor, 2005; Garcillan-Barcia and De la Cruz, 2008). The surface exclusion index (SEI) is defined as the ratio of conjugation efficiency (CE) of matings between [donor cells and plasmid-free recipient cells] and [donor cells and recipient cells of interest]. Thus, stronger exclusion effects are reflected by higher SEIs.

**Table 1:** Evidence that pLS20 contains an exclusion system.

Donor 1	Donor 2	Recipient	Conjugation efficiency	Relative conjugation efficiency
PKS11 (pLS20cat)	–	PS110 (Spec)	1.03⋅10^−3^ (±1.8⋅10^−4^)	1
PKS91 (pLS20spec)	–	PKS7 (Em)	1.80 10^−3^ (±5.0⋅10^−4^)	1.7
PKS11 (pLS20cat)	pKS91 (pLS20spec)	–	8.20 10^−5^ (±5.5⋅10^−6^)	0.08

*Conjugation efficiencies were calculated as transconjugants/donor. Recipient strains PS110 and PKS7 are isogenic except the chromosomally located antibiotic resistance markers, Spec and Em, respectively. Conjugation efficiency values correspond to the mean value of at least three independent experiments. Standard deviations are given in brackets.*

### Gene *29* Is Crucial for pLS20cat Exclusion

To identify the protein(s) responsible for pLS20cat exclusion we performed pBlast and tBlastN searches to find possible similarities between pLS20 (protein) sequences and those of known exclusion system(s). These analyses did not result in the identification of any significant similarity, indicating that pLS20cat contains a hitherto unknown exclusion system. To make the searches more specific we screened all (putative) pLS20cat genes for candidates encoding putative surface proteins, and identified gene *29* (390 codons) and the flanking gene *30* (99 codons) as potential exclusion genes because they were predicted to encode proteins having an N-terminal transmembrane spanning domain but lacking a predicted cleavage site for a signal peptidase. The majority of the p29 protein was predicted to be located at the outside of the cell membrane (see Supplementary Figure S1A), while the majority of the p30 protein was predicted to be located at the inside of the cell membrane (see Supplementary Figure 1B). Based on the genetic organization we considered that both gene *29* and *30* might play a role in exclusion (see Supplementary Figure 1C).

**FIGURE 1:**
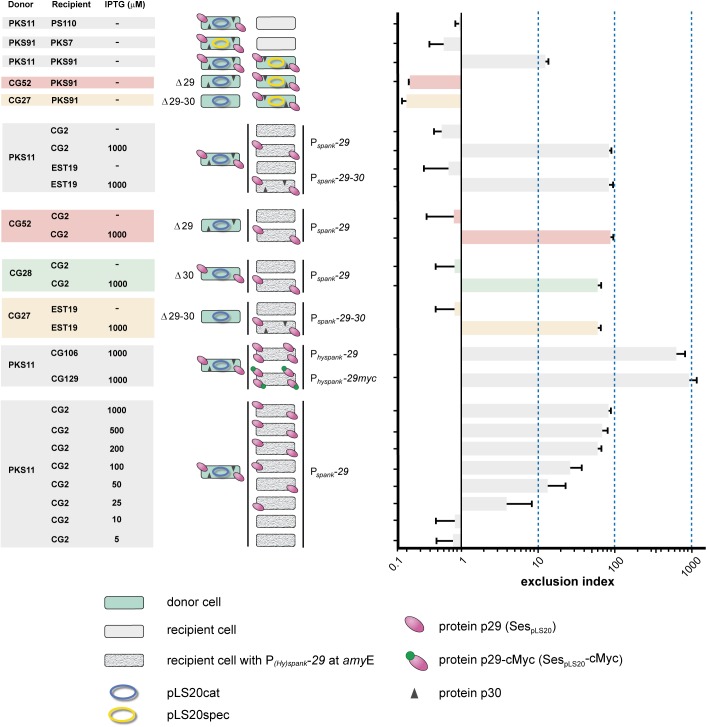
Surface exclusion indexes of matings. Names of donor and recipient strains and induction levels are given on the left. Cartoons of each mating are shown in the middle panel. Donor cells are shown in green, and the plasmid is indicated with an ellipse; blue and yellow representing pLS20cat and pLS20spec, respectively. Deletion derivatives of pLS20cat lacking gene *29*, *30* or both are indicated (Δ29, Δ30, Δ29-30, respectively). Color codes are used to indicate the genotype of the plasmid: Δ29, red; Δ30, green; Δ29-30, orange. Plasmid-free recipient cells are shown in gray, and with a granite pattern if they harbor a chromosomal cassette containing a copy of pLS20cat gene *29*, *30* or both under the control of the IPTG-inducible P_(*hy*)*spank*_ promoter. pLS20cat protein p29 and p30 are schematically indicated with pink ovals and black triangles, respectively. The cMyc tag is indicated with small green oval on top of the protein p29 symbol. Each mating was performed at least three times and the bars correspond to the mean value of these experiments with error bars indicating standard deviations.

To test whether gene *29* and/or *30* are involved in SE we constructed derivatives of pLS20cat lacking either gene *29* (pLS20catΔ29) or both genes (pLS20catΔ29-30), and used strains harboring either plasmid as donor in matings with another donor strain harboring a wild type version of the plasmid. CEs between donor strains CG52 (pLS20catΔ29) and PKS91 (pLS20spec) were about 50-fold higher than those obtained between PKS11 (pLS20cat) and PKS91 (pLS20spec) (see Figure 1). This strongly indicated that gene *29* played a crucial role in SE. In addition, these results demonstrated that the decreased conjugation efficiencies observed in matings between two donor strains harboring a wild type plasmid (pLS20cat/spec) were not due to incompatibility of the transferred plasmid with the resident plasmid in the cell. Finally, it is worth noting that matings between two donor cells allows bi-directional transfer of the plasmid. This probably explains why the CE between donor strains CG52 and PKS91 was about 5-fold higher than the CE obtained in standard matings between a donor and plasmid-free cell.

Conjugation experiments using the double deletion strain CG27 (pLS20catΔ29-30) gave very similar conjugation efficiencies to that of strain CG52 (pLS20catΔ29) when mated with PKS91 (pLS20spec) (see Figure 1), showing that the absence of protein p30 did not inflict an additional defect on the SE system.

### Expression of Gene *29* in Recipient Cells Is Sufficient for Exclusion

To study whether gene *29* is sufficient for SE we constructed strain CG2 in which gene *29* was placed at the chromosomal *amyE* locus under the control of the isopropyl β-D-1-thiogalactopyranoside (IPTG)-inducible P*_spank_* promoter. We also constructed strain EST19 containing both genes *29* and *30* under the control of the P*_spank_* promoter. Next, each of these two strains was employed as the recipient in conjugation experiments using PKS11 (pLS20cat) as donor. As expected, in the absence of IPTG, low SEIs were obtained for matings using CG2 (P*_spank_*-*29*) or EST19 (P*_spank_*-*29*-*30*) as recipient. Importantly, high SEIs were obtained when either recipient strain was grown in the presence of 1 mM IPTG (see Figure 1). These results demonstrate that (i) expression of only gene *29* is sufficient for SE, and (ii) protein p29 is functional in the absence of protein p30.

We next tested whether SE required protein p29 to be present on both mating cells by comparing matings between recipient CG2 cells (P*_spank_*-*29*) ectopically expressing gene *29*, and CG52 donor cells harboring pLS20catΔ29. As shown in Figure 1, high SEIs were obtained for these matings when gene *29* was induced in recipient cells, indicating that SE was not due to interaction between protein p29 molecules on both mating cells.

The results presented in this section and those presented above demonstrate that gene *29* encodes the SE protein of pLS20cat, which henceforth we name *ses_pLS20_* (surface exclusion system pLS20).

### Ses_pLS20_ Is Located on the Cell Surface

**FIGURE 2:**
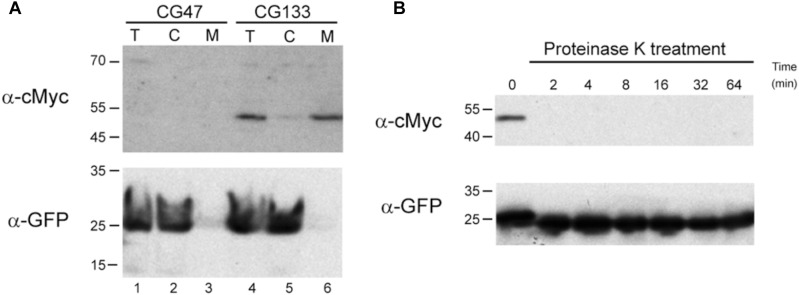
Ses_pLS20_ is located on the cell surface. **(A)** Western blots of fractionated cell samples incubated with antibodies against either the cMyc epitope tag or GFP. Samples of CG47 (*lacA*::P*_xyl_*-*gfp*, lanes 1–3) and CG133 (*amyE*:: P*_hyspank_*-*ses_pLS20_*-*cMyc*, *lacA*::P*_xyl_*-*gfp*, lanes 4–6) cultures growing in the presence of IPTG (1 mM) and xylose (2%) were processed resulting in total (T, lanes 1 and 4), cytosolic (C, lanes 2 and 5), and membrane (M, lanes 3 and 6) fractions. These samples were used for two identically loaded SDS-PAA gels, which were subsequently subjected to immunoblotting using antibodies against either the cMyc epitope tag or GFP. Positions of molecular weight markers (kDa) are given on the left. **(B)** Proteinase K treatment of intact cells expressing *ses_pLS20_-cMyc*. CG133 (*amyE*:: P*_hyspank_*-*ses_pLS20_*-*cMyc*, *lacA*::P*_xyl_*-*gfp*) cells grown in the presence of IPTG and xylose were harvested, resuspended in a proteinase K containing buffer, and incubated at 37°C. One aliquot of this sample was taken immediately (*t* = 0) and other aliquots were withdrawn at the indicated times (min). After processing, these samples were analyzed on two identically loaded SDS-PAA gels, which were subsequently subjected to immunoblotting using antibodies against the cMyc epitope tag or GFP. Positions of molecular weight markers (kDa) are given on the left.

To investigate whether Ses_pLS20_ is a surface protein as predicted we determined the cellular location of a cMyc-tagged version of Ses_pLS20_, named *ses_pLS20_-cMyc*. Thus, we first constructed strain CG129 which contained a copy of *ses_pLS20_-cMyc* (the only copy of *ses_pLS20_*) at the *amyE* locus under the control of the IPTG-inducible P*_hyspank_* promoter. High SEI levels were obtained when the expression of *ses_pLS20_-cMyc* was induced in recipient cells, showing that the fusion protein is functional (Figure 1). We then introduced into strain CG129 a cassette containing the *gfp* gene under the control of the xylose-inducible promoter P*_xyl_* at the *lacA* locus, resulting in strain CG133. The intracellular soluble GFP protein served as an internal control in the experiments described below. To study if Ses_pLS20_ is a membrane protein we prepared total (T), cytosolic (C) and membrane (M) fractions of CG133 cells grown in the presence of IPTG and xylose, and subjected these to SDS-PAGE followed by immunoblotting using antibodies against cMyc. Figure 2A shows that a clear signal corresponding to a protein having an estimated size of ∼48 kDa, which is slightly more than the calculated weight of 43.3 kDa for Ses_pLS20_-cMyc, was obtained for the total protein sample (upper panel, lane 4) of the CG133 cells but not in identically treated samples of the negative control strain CG47 (upper panel, lane 1). A strong signal migrating to the same position was also detected in the membrane fraction of CG133 cells, but only a very minor signal was observed in the cytosolic fraction (upper panel, lanes 6 and 5, respectively). As a control, a duplicate immunoblot was performed with antibodies against GFP. As expected, the vast majority of the GFP signal was obtained in the cytosolic fractions (Figure 2A, lower panel). Together, these results provide compelling evidence that Ses_pLS20_-cMyc is a membrane protein.

To determine whether Ses_pLS20_-cMyc is located at the cell surface we treated intact CG133 cells, grown in the presence of both IPTG and xylose, with proteinase K (protK), and then assessed whether this affected the detection of Ses_pLS20_-cMyc or GFP. The aliquots of the cells treated with protK for different periods of time were lysed and subjected to SDS-PAGE, followed by immunobloting using antibodies against cMyc or GFP. As expected, a clear signal corresponding to Ses_pLS20_-cMyc was detected in the protK-untreated sample (*t* = 0) (Figure 2B, upper panel), but the signal was no longer detected when the samples had been treated for two min or more with protK. On the contrary, strong signals were still observed for GFP even in samples treated for 64 min with protK (Figure 2B, lower panel). These results show that protK degraded the cMyc epitope fused to Ses_pLS20_ but not the cytosolic GFP protein, strongly indicating that Ses_pLS20_-cMyc is located at the surface of the cells.

### Genes *ses_pLS20_* and *30* Are Under the Control of Two Promoters

**FIGURE 3:**
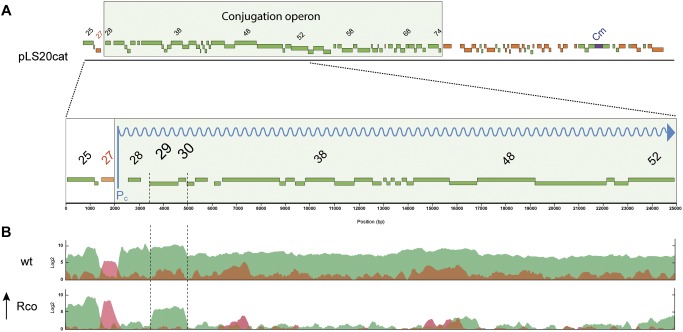
pLS20cat genes *ses_pLS20_* and *30* are expressed independently of the main conjugation promoter P_c_. **(A)** Schematic presentation of the genetic organization of pLS20cat. Position and size of genes are indicated with rectangular bars; green and red colors present forward and reverse orientation of the genes, respectively. The conjugation operon is boxed. The chloramphenicol resistance gene (Cm) is indicated in blue. A blow-up of the region spanning genes *25* to *52* is shown in the lower part. The reversely oriented gene *27* encodes the transcriptional regulator Rco_pLS20_. **(B)** Expression profiles of pLS20cat genes determined by RNAseq. Total RNA was isolated from late exponentially growing cells of strains PKS11 (pLS20cat; upper panel) and PKS14 (P*_spank_*-*rco_pLS20_*, pLS20cat, lower panel) grown in the presence of 1 mM IPTG. Expression levels are presented on a log2 scale. Forward and reversely oriented transcripts are indicated in green and red, respectively. Note that *rco_pLS20_* (gene *27*) was expressed at a higher level in PKS14 than in PKS11 cells due to the additional induced expression of this gene from the ectopic locus at *amyE*. Results corresponding to pLS20cat genes *25* till *52* are shown. Data presented here are from a newly executed RNAseq experiment. Supplementary Figure S2 shows that a very similar expression profile was obtained using the data of the previously published RNAseq experiment (Singh et al., 2013).

**FIGURE 4:**
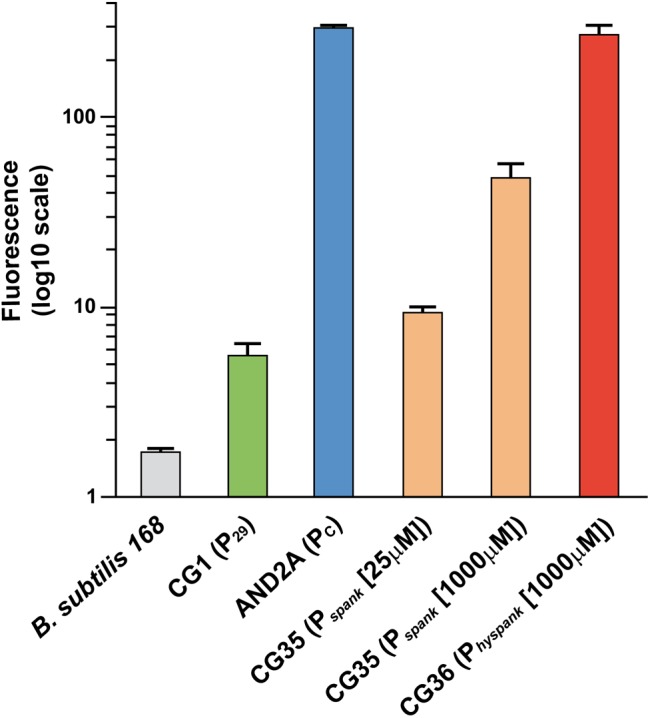
Relative promoter strength determined by FACS analysis of strains containing a transcriptional *gfp* fusion. Strains having at the *amyE* locus a cassette containing a single copy of the *gfp* reporter gene fused to a promoter of interest, and a negative control strain were used for FACS analysis at an OD_600_ = 1. At least 100,000 cells were analyzed for each sample. Strains: 168, gray; CG1 (P*_*29*_*-*gfp*), green; AND2A (P*_c_*-*gfp*), blue; CG35 (P*_spank_*-*gfp*) grown in the presence of 25 or 1,000 μM IPTG, orange; CG36 (P*_hyspank_*-*gfp*) grown in the presence of 1 mM IPTG, red. For each strain the mean values of geomean determinations of at least three independent FACS analyses are given together with their standard deviations.

To carry out their function, exclusion proteins are expected to be expressed constitutively. However, *ses_pLS20_* is located within the conjugation operon of pLS20cat that is controlled by the main conjugation promoter P*_c_* (Singh et al., 2013, see Figure 3A). This promoter is strictly repressed except during a transient period of time when an anti-repressor relieves the repression (Singh et al., 2013; Ramachandran et al., 2014). We therefore re-evaluated our previous RNAseq data, obtained from pLS20cat-containing cell samples with/out ectopic overexpression of the repressor of promoter P*_c_*, Rco_pLS20_ (Singh et al., 2013), which showed that all the conjugation genes were repressed most of the time. Previously, a heatmap presentation was used in which color codes indicate differential gene expression under both conditions. Since this might obscure possible expression from a promoter different from P*_c_* we plotted the levels of expression along the plasmid genome for either condition, using the RNAseq data of a newly executed experiment (Figure 3) as well as those of the experiment published before (Singh et al., 2013, Supplementary Figure S2). As expected, genes in the conjugation operon were expressed at high and low levels without and with ectopic *rco_pLS20_* induction, respectively. However, substantial levels of expression were still observed for genes *ses_pLS20_* and *30* when *rco_pLS20_* was induced, raising the possibility that genes *ses_pLS20_* and *30* were expressed from another promoter that is not controlled by Rco_pLS20_. To test this, we cloned the *ses_pLS20_* upstream region in front of a *gfp* reporter gene (see Materials and Methods; Rudge et al., 2013) and placed it at the chromosomal *amyE* locus (strain CG1). Using the same strategy, we also constructed strain AND2A, in which the *gfp* gene is controlled by the strong P*_c_* promoter (Ramachandran et al., 2014). FACS analysis of late exponential cells (OD_600_ = 1) was then used to determine fluorescence levels (Figure 4). As expected, a high level of fluorescence was observed for strain AND2A (P*_c_*-*gfp*). Importantly, although lower than that observed for AND2A, CG1 cells were also fluorescent, demonstrating that the cloned region upstream of the *ses_pLS20_* gene contains a promoter, which we name P*_*29*_*. Fluorescence levels of strains CG35 and CG36 are explained below.

### Gene *30* Is Not Involved in the Exclusion System of pLS20cat

Although gene *30* is co-transcribed with *ses_pLS20_* from the P_*29*_ promoter, results presented above did not indicate that it had a role in exclusion. To study a possible role of gene *30* in more detail we constructed strain CG28 that harbored a derivative of pLS20cat lacking gene *30* (pLS20catΔ30, see Materials and Methods) and used it as donor in matings with a plasmid-free recipient strain expressing *ses_pLS20_* (i.e., CG2). The absence of gene *30* did not significantly affect CE (Figure 1). This, together with the results presented above, demonstrates that gene *30* does not play a crucial role in SE.

### Correlation Between the Level of Surface Exclusion and *ses_pLS20_* Induction

Induction of *ses_pLS20_* in recipient cells from the P*_spank_* promoter in the presence of 1 mM IPTG provoked SE (Figure 1). Interestingly, in this experimental set up the SEI was about 10-fold higher than that observed for matings between two donor strains. This discrepancy might be due to different *ses_pLS20_* expression levels in the two experimental set-ups. To test this, we performed matings in which *ses_pLS20_* was induced to different levels in the recipient strain CG2. The results presented in Figure 1 show a direct correlation between the extent of SE and the level of gene *ses_pLS20_* induction. Next, we expressed *ses_pLS20_* in recipient cells from a promoter stronger than P*_spank_*, the IPTG-inducible P*_hyspank_* promoter. The strain, CG106, was used as a recipient strain in matings with PKS11 (pLS20cat) as donor strain. When grown in the presence of 1 mM IPTG, the SEI value obtained for strain CG106 (P*_hyspank_*-*ses_pLS20_*) was about 10-fold higher than that obtained for strain CG2 (P*_spank_*-*ses_pLS20_*) and about 100-fold higher than that obtained for matings between two donor strains (see Figure 1).

To compare the (maximum) strengths of promoters P*_*29*_*, P*_c_*, P*_spank_*, and P*_hyspank_* we constructed strains CG35 and CG36 containing a cassette in which *gfp* was placed under the control of the P*_spank_* and the P*_hyspank_* promoter, respectively, and determined the fluorescence levels of these cells growing in the presence of 1 mM IPTG in parallel with those of strains CG1 (P*_*29*_*-*gfp*) and AND2A (P*_c_*-*gfp*) (Figure 4). The results obtained confirmed that the maximum strength of P*_hyspank_* promoter was considerably stronger (by at least 6-fold) than that of the P*_spank_* promoter. In addition, the results showed that P*_*29*_* was the weakest of these promoters, and that the strength of P*_c_* was similar to that of the P*_hyspank_* promoter induced to maximum levels. Taken together, these results provide strong evidence that the amount of protein Ses_pLS20_ determines the strength of the SE system.

Although the maximum strength of P*_hyspank_* was similar to that of the P*_c_* promoter (Figure 4), the SEI of matings between two donor strains was much lower than that in matings using recipient cells expressing *ses_pLS20_* from the P*_hyspank_* promoter at 1 mM IPTG. These results could be explained bearing in mind that the P*_c_* promoter became activated only in a subpopulation of the donor cells (Rosch and Graumann, 2015). The modest SEI levels observed for matings between two donor strains would therefore be the consequence of the low-level *ses_pLS20_* expression from the weak P*_*29*_* promoter in receptive donor cells that have not activated the conjugation promoter P*_c_*. This conclusion was supported by the observations that the SEI obtained between two donor strains was similar to that between a donor strain and recipient strain CG2 (P*_spank_*-*ses_pLS20_*) grown in the presence of 25 μM IPTG (Figure 1), and that the strength of promoter P*_*29*_* was similar to that of the P*_spank_* promoter induced at 25 μM IPTG (Figure 4).

### Ses_pLS20_ Is the Founding Member of a New Family of Surface Exclusion Proteins Whose Homologs Are Present in a Number of Firmicutes Bacteria Including Pathogens

*Bacillus pumilus* strain NRS576 harbors a plasmid, p576, which is related to pLS20: they share a similar origin of replication and plasmid segregation module, and they both have a conjugation operon (Singh et al., 2010; Val-Calvo et al., 2019). Based on this we analyzed whether p576 contained a homologue of *ses_pLS20_*. This analysis revealed that the second gene of its putative conjugation operon, gene *38*, a predicted membrane protein that shares significant similarity with Ses_pLS20_ (51% similarity; see Supplementary Figure S3). Based on the similarity and its conserved genetic location within the conjugation operon it was likely that p576 gene *38* encoded a homologue of Ses_pLS20_ which we tentatively named *ses_p576_*. To study whether public databases contained genes encoding proteins similar to Ses_pLS20_/Ses_p576_ we used these protein sequences to perform ‘blastp’ searches. After removing redundant sequences these searches resulted in the identification of 30 hits showing significant similarity to Ses_pLS20_ (see Supplementary Table S4). All the identified genes, which were annotated as unknown function, could encode a putative protein of similar size to Ses_pLS20_. Interestingly, all these hits corresponded to putative genes present in the phylum Firmicutes genera Bacilli, Listeria or Clostridia. Results of additional analyses supported the view that the identified genes encoded homologs of Ses_pLS20_. First, all except one were predicted with more than 95% confidence to encode a protein containing an N-terminal membrane anchor. In the case of the single exception, an N-terminal transmembrane spanning domain was predicted with 60% confidence. In some cases, an analysis of its surrounding context was limited because the identified gene was located at the beginning of a (small) contig. Nevertheless, in 24 out of the 30 cases it could be established that the identified gene formed part of a likely conjugation operon, and in most of these cases the putative conjugation operons was located on a plasmid. Strikingly, the *ses_pLS20_* homologs were one of the first genes of their corresponding putative conjugation operon, as in pLS20 and p576. In summary, our data provide compelling evidence that Ses_pLS20_ constitutes the founding member of a family of SE proteins that forms part of a conjugative element that is present in a subset of bacteria belonging to the Gram+ phylum Firmicutes.

Finally, we also used an *in silico* approach to analyze whether the genes downstream of the identified *ses_pLS20_* homologs shared similarity with pLS20cat gene *30*. As shown in Supplementary Table S5, only the protein encoded by the gene downstream of the *ses_pLS20_* homolog, present on an unnamed putative conjugative *Bacillus atrophaeus* plasmid, shared more than 20% identity with pLS20cat encoded protein p30. This further supports the view that the identified SE system is composed of the single *ses_pLS20_*-like gene.

## Discussion

Probably all conjugative plasmids contain at least one exclusion gene (Garcillan-Barcia and De la Cruz, 2008). Improving our knowledge about exclusion systems may open ways to impede or block conjugation-mediated spread of antibiotic resistance. Most information available on exclusion systems is related to conjugative plasmids replicating in G− bacteria, prompting us to study the SE system of the Gram+ *B. subtilis* conjugative plasmid pLS20. Taking into account the large differences in the cell wall organization between G− and G+ bacteria, the SE proteins encoded by conjugative elements replicating in G+ bacteria may be very different from those replicating in G− bacteria. The little information that is available indicates that this is indeed the case. As far as we know, the only genuine G+ exclusion systems described are those encoded by the so-called enterococcal conjugative sex-pheromone plasmids, particularly those present on pCF10 and pAD1 (Dunny et al., 1985; Kao et al., 1991; Weidlich et al., 1992). These plasmids contain a conserved gene, *sec*10 (a.k.a. *prg*A) or *sea*1 encoding a surface-located protein that is responsible for SE (Clewell and Brown, 1980; Dunny et al., 1985).

Here we have demonstrated that pLS20 contains a SE system that is different from all exclusion systems described so far, and that homologues of *ses_pLS20_* are present on other (likely) conjugative plasmids of Firmicutes bacteria including (opportunistic) pathogens like Listeria or Clostridia.

It is generally believed that the primary function of exclusion systems is to prevent redundant transfer of conjugative elements. However, matings between two pLS20-containing donor strains revealed that, although redundant transfer was inhibited, it was far from being blocked. As outlined in more detail below, this is also the case for most if not all SE systems studied so far and indicates that the function of exclusion systems is more complex than “simply” preventing redundant transfer of a conjugative element.

**FIGURE 5:**
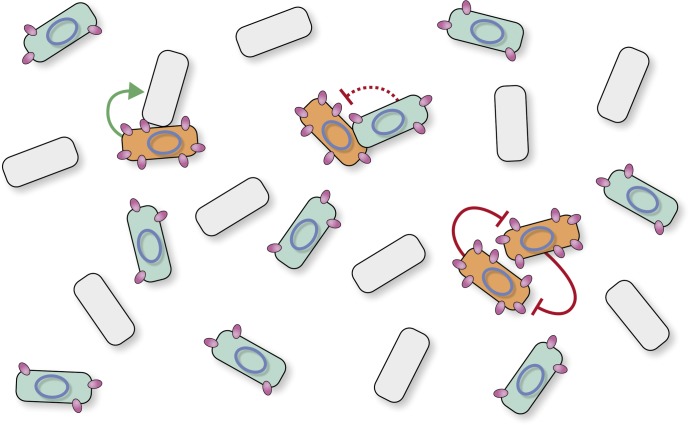
Model depicting different surface exclusion levels for conjugative transfers between two conjugation-primed donor cells, and between conjugation-primed donor cells with non-primed donor cells. Cartoon of a mixed population of cells consisting of plasmid-free recipients (shown in gray) and pLS20-containing donor cells (cells with a blue ellipse). Donor cells express low levels of Ses_pLS20_ (shown as small purple ovals) due to the weak constitutive P_*29*_ promoter (cells shown in blue). However, because *ses_pLS20_* is also under the control of the strong conjugation promoter P*_c_*, its expression becomes up-regulated in the subpopulation of donor cells that have activated the conjugation genes (shown as orange cells). The extent of surface exclusion is directly related to the level of *ses_pLS20_* expression. Conjugation is not inhibited between conjugation-primed donor and plasmid-free recipient cells that do not express Ses_pLS20_ (reflected with green solid line arrow), resulting therefore in optimal conjugation efficiencies and maximum spread of conjugative plasmid in the population. Low-level expression of Ses_pLS20_ in non-conjugation primed donor cells moderately inhibits conjugative transfer between [conjugation-primed and non-conjugation-primed donor cells] (indicated with interrupted red line ending in a “T” shape) favoring efficient spread of the plasmid in the population, as well as genetic exchange (i.e., evolutionary adaptation) between genes and/or modules associated with the conjugation module. Finally, high levels of Ses_pLS20_ expression efficiently inhibit conjugation between two conjugation-primed donor cells (indicated with solid red lines ending in a “T”-shape).

The major findings of these studies can be summarized as follows: (i) *ses_pLS20_* is the only necessary gene to exert exclusion, (ii) there is a direct relation between the level of *ses_pLS20_* expression and the level of exclusion, (iii) *ses_pLS20_* is differentially expressed in donor cells having or not activated the conjugation pathway; i.e., *ses_pLS20_* is expressed at low basal levels in all donor cells from the weak promoter P*_*29*_* that is located immediately upstream of *ses_pLS20_*, but it is expressed to much higher levels in conjugation-primed cells due to the additional expression from the strong conjugation promoter P*_c_*. Whereas the basal expression level of *ses_pLS20_* in all donor cells inhibits redundant plasmid transfer only moderately (∼10-fold), its high-level expression in conjugation-primed cells inhibits redundant plasmid transfer very efficiently (∼1,000-fold). Thus, as schematically shown in Figure 5, our results revealed that the SE system of pLS20 has a dual effect: it inhibits moderately the transfer of the plasmid between donor cells in general, but efficiently prevents the transfer of pLS20 between two conjugation-primed cells.

The fact that a sophisticated transcriptional circuitry has evolved to differentially express *ses_pLS20_* in primed and non-primed donor cells suggests that the resulting differences in SE levels in these cells underlie evolutionary advantage(s). For instance, inhibition of plasmid transfer between two donor cells will contribute to more efficient spreading of the plasmid to cells not harboring the plasmid. However, by preventing redundant transfer only partially it also may contribute to genetic flexibility of the plasmid and the host. For this it has to be taken into account that plasmids have a modular organization; the essential backbone of a plasmid, -comprising the replication and maintenance functions-, is generally combined with one or more non-essential modules, including a conjugation module, which can be interchanged with other (extra)chromosomal DNA residing in the bacterium (for review see, Frost et al., 2005; Norman et al., 2009). Therefore, moderate inhibition of redundant plasmid transfer is beneficial for the plasmid and their hosts in evolutionary terms: it favors efficient spreading of the plasmid in a population of cells, without losing the ability to exchange modules or genes that are associated with another conjugative element sharing the same SE system. Probably this is also the reason why the SE gene is embedded within the conjugation operon. If located outside of the conjugation operon, it can become uncoupled from the conjugation operon if such a plasmid recombines with another (conjugative) DNA element. A conjugation module lacking a SE system will be compromised in efficient spreading of the plasmid and be outcompeted by an element containing a SE system.

Transfer of plasmids between two conjugation-primed donor cells, on the other hand, is efficiently inhibited. In addition to preventing futile plasmid exchanges, the other reason for the strong inhibition could be that simultaneous bidirectional transfer is not compatible. The transfer process involves the establishment of a mating pair, local lysis of the cell wall, and the formation of a sophisticated membrane-associated translocation machinery that connects the mating cells and transfers – besides some proteins – many kbp of DNA (∼65 kb in the case of pLS20) in its single-stranded form. It is conceivable that simultaneous bidirectional transfer of the plasmid is not feasible due to, for instance, incompatibility of the translocation machineries with opposing directionalities and/or incompatibility of the incoming ssDNA with the simultaneous production of ssDNA to be transferred.

Similar to what we observed for pLS20, all SE systems studied so far, regardless whether present on ICEs or conjugative plasmids of G− or G+ origin, do not efficiently inhibit conjugation when analyzed at population level, with a inhibition range of 10 to 20-fold (Clewell and Brown, 1980; Dunny et al., 1985) (for review see Garcillan-Barcia and De la Cruz, 2008). Furthermore, it appears that most if not all SE genes are located within the conjugation operon. This applies to exclusion genes present in ICEs as well as conjugative plasmids of G− or G+ origin. Therefore, the particular structural organization in which the SE gene is embedded in the conjugation operon, and the differential transcriptional expression of the SE gene in non-conjugation-primed and conjugation-primed donor cells appears to be a common feature of SE systems.

In summary, besides unraveling a novel SE system present on pLS20, our results permitted the identification of putative similar systems on other conjugative elements present in Gram+ bacteria and adds a new dimension to the function of SE systems in general.

## Data Availability

RNAseq data are deposited in the SRA repository (submission SUB5726602).

## Author Contributions

All authors have made substantial, direct experimental and/or intellectual contribution to the work. CG-C, JV-C, AM-A, ES, and PS generated all plasmids and strains, purified proteins, and conducted all the experiments. DA and JV-C carried out *in silico* analyses. DA also contributed to the general design and analyses of the results. LW and WM designed the experimental plan and were principally responsible for analyzing the results and writing the manuscript. WM supervised the contributions of CG-C, JV-C, AM-A, ES, and PS.

## Conflict of Interest Statement

The authors declare that the research was conducted in the absence of any commercial or financial relationships that could be construed as a potential conflict of interest.

## Footnotes

1 www.cbs.dtu.dk/servises/TMHMM

2 http://www.cbs.dtu.dk/services/SignalP/

3 https://blast.ncbi.nlm.nih.gov/Blast.cgi?PAGE=Proteins

4 https://www.drive5.com/usearch/
