# Supplementary material for: Surface Exclusion Revisited: Function Related to Differential Expression of the Surface Exclusion System of Bacillus subtilis Plasmid pLS20
Source: Front Microbiol. 2019 Jul 10;10:1502. doi: 10.3389/fmicb.2019.01502 (PMC6635565; doi:10.3389/fmicb.2019.01502)
Supplement: Supplementary file 1 [file Image_1.pdf]

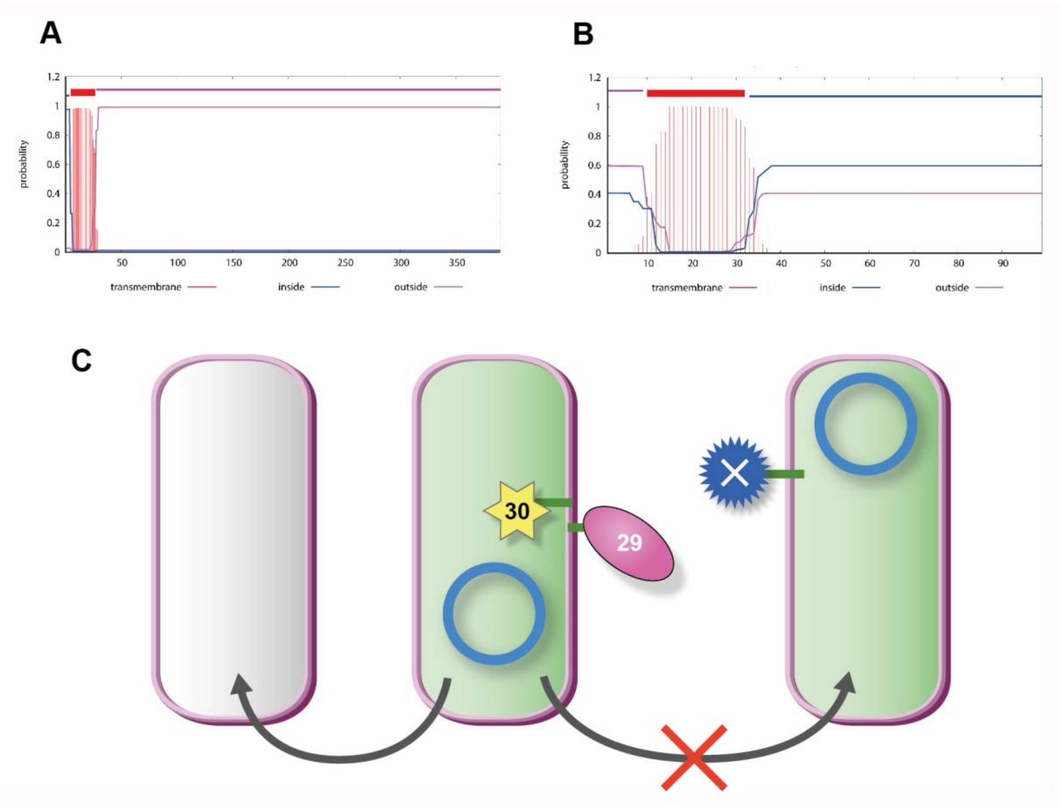

Supplemental Figure S1. Identification of pLS20cat genes *29-30* as the putative surface exclusion genes, and working model of the pLS20cat surface exclusion mechanism based on predicted features of proteins p29 and p30. A-B. Prediction of transmembrane helices (red) and membrane topology of pLS20cat-encoded proteins p29 (A, 390 residues) and p30 (B, 99 residues) according a hidden Markov model (see Materials and Methods, Sonnhammer et al., 1998; Krogh et al., 2001).

C. Working model of the pLS20cat SE system. Conjugation is inhibited between two donor cells (indicated in green) but not between a donor (middle green cell) and recipient cell (left grey cell). Inhibition of conjugation is proposed to be due to interaction of a surface-located protein present on a receptive donor cell (indicated as ”X”) with pLS20cat protein p29 located on the surface of the conjugation-primed donor cell. Protein p30 might be involved in exclusion; for instance, by stabilizing protein p29 or by triggering a signal to the cytosol to inhibit conjugation. Blue circle: pLS20cat. Transmembrane spanning domains are indicated with short green rectangles.

References

Krogh, A., Larsson, B., von, H.G., and Sonnhammer, E.L. (2001). Predicting transmembrane protein topology with a hidden Markov model: application to complete genomes. *J. Mol. Biol* 305(3), 567-580. doi: 10.1006/jmbi.2000.4315 [doi];S0022-2836(00)94315-8 [pii].

Sonnhammer, E.L., von Heijne, G., and Krogh, A. (1998). "A hidden Markow model for predicting transmembrane helices in protein sequences," in *Proc. Int. Conf. on Intelligent Systems for Molecular Biology*, eds. J. Glasgow, T. Littlejohn, F.

Major, R. Lathrop, D. Sankoff & C. Sensen. 1 ed (Menlo Park, CA: AAAI press), 175-182.
