# Supplementary material for: Surface Exclusion Revisited: Function Related to Differential Expression of the Surface Exclusion System of Bacillus subtilis Plasmid pLS20
Source: Front Microbiol. 2019 Jul 10;10:1502. doi: 10.3389/fmicb.2019.01502 (PMC6635565; doi:10.3389/fmicb.2019.01502)
Supplement: Supplementary file 2 [file Image_2.pdf]

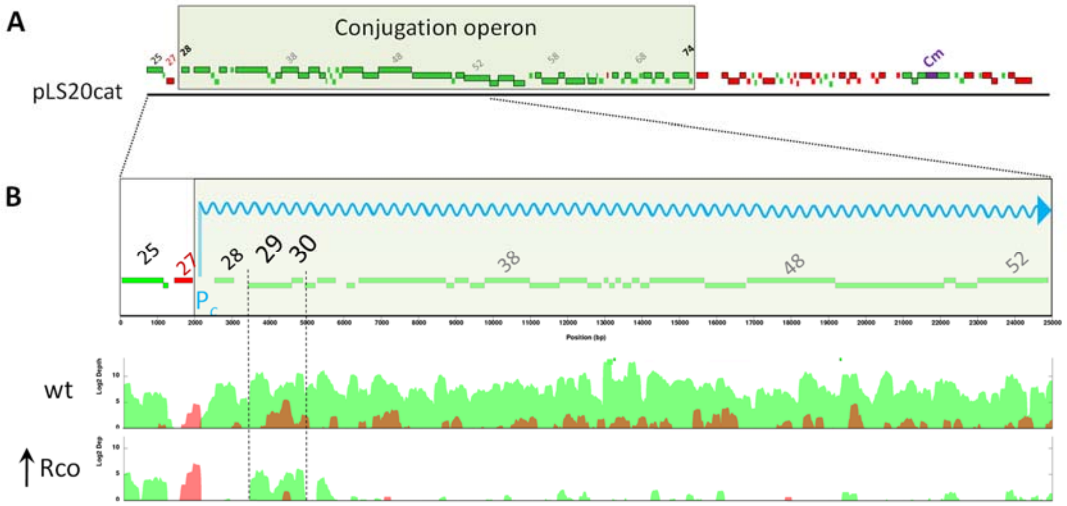

Supplemental Figure S2. pLS20cat genes *29* and *30* are expressed independently of the main conjugation promoter P_c. A. Schematic presentation of the genetic organization of pLS20cat. Position and size of genes are indicated with bars; green and red colors present forward and reverse orientation of the genes, respectively. The conjugation operon spanning genes *28* till *74* are boxed. The chloramphenicol resistance gene, which is inserted at the unique *Sal*I site, is indicated (Cm) and shown in blue. A blow-up of the region spanning genes *25* to *52* is shown in the lower part. The reversely oriented gene *27* encodes the transcriptional regulator Rco_{pLS20}.

B. Expression profiles of pLS20cat genes determined by RNAseq. Total RNA was isolated from late exponentially growing cells when conjugation efficiency is at its maximum of strains PKS11 (pLS20cat; upper panel) and PKS14 (P_{spank}_rco_{pLS20}, pLS20cat, lower panel). RNAseq data correspond to an experiment for which the results were published before using a different format: differential heatmap presentation (Singh et al., 2013). Expression levels are presented on a log2 scale. Forward and reversely oriented transcripts are indicated in green and red, respectively. Note that rco_{pLS20} (gene *27*) was expressed at a higher level in PKS14 than in PKS11 cells, which is due to the additional induced expression of this gene from the ectopic locus at *amyE*. Results corresponding to pLS20cat genes *25* till *52* are shown.

References

Singh, P.K., Ramachandran, G., Ramos-Ruiz, R., Peiro-Pastor, R., Abia, D., Wu, L.J., et al. (2013). Mobility of the Native Bacillus subtilis Conjugative Plasmid pLS20 Is Regulated by Intercellular Signaling. *PLoS Genet* 9(10)**,** e1003892. doi: 10.1371/journal.pgen.1003892 [doi];PGENETICS-D-13-01403 [pii].
