# Supplementary material for: Surface Exclusion Revisited: Function Related to Differential Expression of the Surface Exclusion System of Bacillus subtilis Plasmid pLS20
Source: Front Microbiol. 2019 Jul 10;10:1502. doi: 10.3389/fmicb.2019.01502 (PMC6635565; doi:10.3389/fmicb.2019.01502)
Supplement: Supplementary file 3 [file Image_3.pdf]

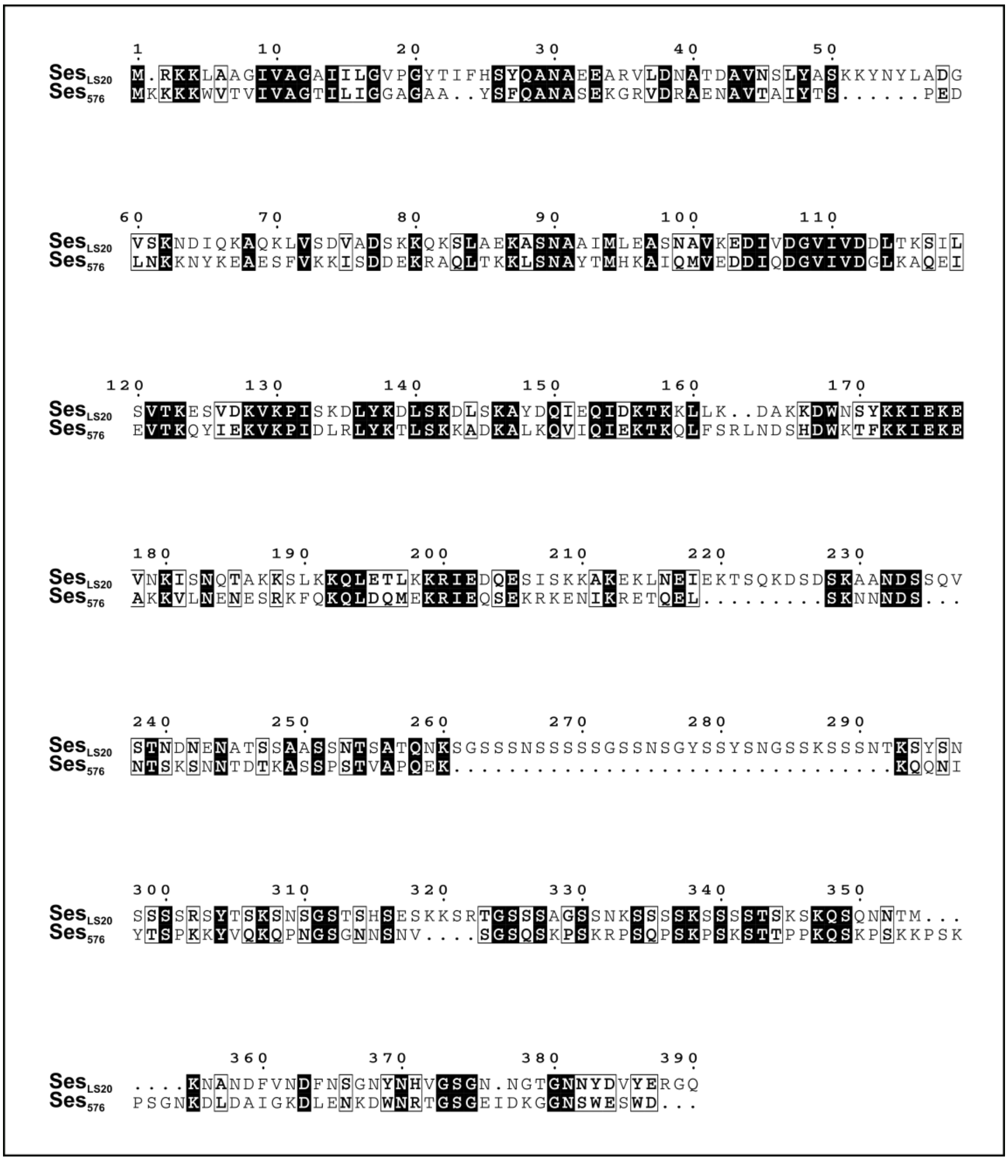

Supplemental Figure S3. Alignment of the deduced protein sequences of pLS20cat gene *29* and p576 gene *38*. The alignment was performed using the MAFFT server (http://mafft.cbrc.jp/alignment/server/index.html) applying the following strategy (E-INS-i, not aligning gapped regions) and parameters (Blosum 62 scoring matrix). The following residues were considered conservative: HKR (polar positive); DE (polar negative); STNQ (polar neutral); AVLIM (non-polar aliphatic); FYW (non-polar aromatic).
