# Supplementary material for: Surface Exclusion Revisited: Function Related to Differential Expression of the Surface Exclusion System of Bacillus subtilis Plasmid pLS20
Source: Front Microbiol. 2019 Jul 10;10:1502. doi: 10.3389/fmicb.2019.01502 (PMC6635565; doi:10.3389/fmicb.2019.01502)
Supplement: Supplementary file 4 [file Table_1.docx]

| **Supplemental Table S1.** Strains used | | | |
| --- | --- | --- | --- |
| ***Strains*** | | **Genotype or description** | **Reference or source** |
| *E. coli* | |  |  |
|  | XL1-Blue | *end*A1 *gyr*A96(nal^R^) *thi*-1 *rec*A1 *rel*A1 *lac* *gln*V44 F'[ ::Tn10 *pro*AB^+^ *lac*I^q^ Δ(*lac*Z)M15] *hsd*R17(r_K_^-^ m_K_^+^) | (Bullock et al., 1987) |
|  | JM101 | *gln*V44 *thi*-1 Δ(l*ac*-*pro*AB) F'[*lac*I^q^ZΔM15 *tra*D36 *pro*AB^+^] (*rec*A^+^, r_K_^+^) | (Yanish-Perron et al., 1985) |
|  | ECE74 | strain JM103 harboring plasmid pCm::Sp | (Steinmetz and Richter, 1994); BGSC* |
| *B. subtilis* | |  |  |
|  | 168 (1A700) | *trp*C2 | BGSC |
|  | PS110 | *trp*C2*, amyE::*P*_spank_-Δ (spec)* | (Singh et al., 2013) |
|  | PKS7 | *trpC2*, *thrC*::*em* | (Singh et al., 2013) |
|  | PKS11 | *trpC2*, pLS20cat | (Singh et al., 2012) |
|  | PKS14 | *trpC2*, *amyE*::P*_spank_*-*rco_pLS20_* (*spec*), pLS20cat | (Singh et al., 2013) |
|  | PKS56 | BGSC strain 1A976 (*his* *npr*E18 *apr*E3 *egl*S Δ102 *bgl*T/*bgl*S ΔEV *lac*A::P*_xyl_*-*comK* (*em*)) harboring pLS20cat | (Singh et al., 2012) |
|  | PKS76 | PKS56 derivative harboring pLS20spec | This work |
|  | PKS91 | *trpC2,* pLS20spec | This work |
|  | AND2A | *trpC2, amyE::*P*_C_*-*gfp* (cat) | This work |
|  | EST19 | *trpC2, amyE::* P*_spank_-29-30* (*spec*) | This work |
|  | CG1 | *trpC2, amyE::*P*_29_*-*gfp* (*cat*) | This work |
|  | CG2 | *trpC2, amyE::* P_spank_-*ses_pLS20_* (*spec*) | This work |
|  | CG3 | *trpC2, amyE::* P*_spank_-30* (*spec*) | This work |
|  | CG27 | *trpC2,* pLS20catΔ29-30 | This work |
|  | CG28 | *trpC2,* pLS20catΔ30 | This work |
|  | CG35 | *trpC2, amyE::*P*_spank_*-*gfp* (*spec*) | This work |
|  | CG36 | *trpC2,* *amyE::*P*_hyspank_-gfp* (*spec*) | This work |
|  | CG47 | *trpC2, lacA*::P*_xyl_*-*gfp* (*em*) | This work |
|  | CG52 | *trpC2,* pLS20catΔ29 | This work |
|  | CGEST19 | *trpC2, amyE::* P_hyspank_-*29-30* (*spec*) | This work |
|  | CG106 | *trpC2, amyE::* P_hyspank_-*29* (*spec*) | This work |
|  | CG129 | *trpC2, amyE::* P_hyspank_-*ses_pLS20_-cMyc_LS20_* (*spec*) | This work |
|  | CG133 | *trpC2, amyE::* P_hyspank_-*ses_pLS20_-cMyc_LS20_* (*spec*), *lacA*::P*_xyl_*-*gfp* (*em*) | This work |

Reference List

1. Bullock WO, Fernandez JM, Short JM (1987) XL1-blue: a high efficiency plasmid transforming *recA* *Escherichia coli* strain with Beta-galactosidase selection. Biotechniques 5: 376-379.

2. Yanish-Perron C, Vieira J, Messing J (1985) Improved M13 phage cloning vectors and host strains: nucleotide sequence of the M13mp18 and pUC19 vectors. Gene 33: 103-119.

3. Steinmetz M, Richter R (1994) Plasmids designed to alter the antibiotic resistance expressed by insertion mutations in *Bacillus subtilis*, through in vivo recombination. Gene 142: 79-83.

4. Singh PK, Ramachandran G, Ramos-Ruiz R, Peiro-Pastor R, Abia D, Wu LJ, Meijer WJ (2013) Mobility of the Native Bacillus subtilis Conjugative Plasmid pLS20 Is Regulated by Intercellular Signaling. PLoS Genet 9: e1003892. 10.1371/journal.pgen.1003892 [doi];PGENETICS-D-13-01403 [pii].

5. Singh PK, Ramachandran G, Duran-Alcalde L, Alonso C, Wu LJ, Meijer WJ (2012) Inhibition of Bacillus subtilis natural competence by a native, conjugative plasmid-encoded comK repressor protein. Environ Microbiol 14: 2812-2825. 10.1111/j.1462-2920.2012.02819.x [doi].

Bullock, W.O., Fernandez, J.M., and Short, J.M. (1987). XL1-blue: a high efficiency plasmid transforming *recA* *Escherichia coli* strain with Beta-galactosidase selection. *Biotechniques* 5**,** 376-379.

Singh, P.K., Ramachandran, G., Duran-Alcalde, L., Alonso, C., Wu, L.J., and Meijer, W.J. (2012). Inhibition of Bacillus subtilis natural competence by a native, conjugative plasmid-encoded comK repressor protein. *Environ. Microbiol* 14(10)**,** 2812-2825. doi: 10.1111/j.1462-2920.2012.02819.x [doi].

Singh, P.K., Ramachandran, G., Ramos-Ruiz, R., Peiro-Pastor, R., Abia, D., Wu, L.J., et al. (2013). Mobility of the Native Bacillus subtilis Conjugative Plasmid pLS20 Is Regulated by Intercellular Signaling. *PLoS Genet* 9(10)**,** e1003892. doi: 10.1371/journal.pgen.1003892 [doi];PGENETICS-D-13-01403 [pii].

Steinmetz, M., and Richter, R. (1994). Plasmids designed to alter the antibiotic resistance expressed by insertion mutations in *Bacillus subtilis*, through in vivo recombination. *Gene* 142**,** 79-83.

Yanish-Perron, C., Vieira, J., and Messing, J. (1985). Improved M13 phage cloning vectors and host strains: nucleotide sequence of the M13mp18 and pUC19 vectors. *Gene* 33**,** 103-119.
