# Supplementary material for: Surface Exclusion Revisited: Function Related to Differential Expression of the Surface Exclusion System of Bacillus subtilis Plasmid pLS20
Source: Front Microbiol. 2019 Jul 10;10:1502. doi: 10.3389/fmicb.2019.01502 (PMC6635565; doi:10.3389/fmicb.2019.01502)
Supplement: Supplementary file 5 [file Table_2.docx]

| **Supplemental Table S2.** plasmids used | | | |
| --- | --- | --- | --- |
| **Plasmids** | | **Description** | **Reference or source** |
|  | pDR110 | *B. subtilis amyE* integration vector containing IPTG-inducible P*_spank_* promoter | gift of David Rudner |
|  | pDR111 | *B. subtilis amyE* integration vector containing IPTG-inducible P*_hyperspank_* promoter | gift of David Rudner |
|  | pCm::Sp | *E. coli* vector designed to replace chloramphenicol resistance gene by spectinomycin resistance gene | (Steinmetz and Richter, 1994); BGSC |
|  | pLS20cat | Native plasmid pLS20 labeled with Cm resistance gene in unique *Sal*I site | (Itaya et al., 2006) |
|  | pLS20spec | Plasmid pLS20 labeled with spectinomycin resistance gene | This work |
|  | pLS20catΔ29 | derivative of pLS20cat in which gene *29* is deleted | This work |
|  | pLS20catΔ30 | derivative of pLS20cat in which gene *30* is deleted | This work |
|  | pLS20catΔ29-30 | derivative of pLS20cat in which genes *29* and *30* are deleted | This work |
|  | pMiniMAD2 | Plasmid used for marker less deletions | gift of Daniel Kearns |
|  | pTA | pTA cloning vector pTZ57R/T | ThermoScientific (USA) |
|  | pAX01 | *B. subtilis lacA* integration vector containing xylose-inducible P*_xyl_* promoter | (Hartl et al., 2001), BGSC |
|  | pTAP28Hind_B | pTA derivative containing 538 bp fragment containing main conjugation promoter P*_c_* (PCR fragment primer set [Prom28UP_Hind - Prom28Dn_Bam]) | This work |
|  | pJS104 | *B. subtilis* *amyE* integration vector containing *sfGFP* gene preceded by a 124 bp *Eco*RI fragment containing the constitutive promoter of bacteriophage SPO1 gene *26*. | Gift of PJ Steiner (UK) (Rudge et al., 2013) |
|  | pKSsfGFP | *B. subtilis amyE* integration vector containing promoter less sf*GFP* gene | This work |
|  | pCG1 | 358 bp upstream region of pLS20cat gene *29* cloned in front of the *gfp* reporter gene present on integration vector pKSsfGFP | This work |
|  | pAND2A | 583 bp pLS20cat region containing the P*_c_* promoter cloned in front of the *gfp* reporter gene present on integration vector pKSsfGFP | This work |
|  | pCG2 | gene *ses_LS20_* cloned behind the P*_spank_* promoter present on pDR110 | This work |
|  | pCG3 | pLS20cat gene *30* cloned behind the P*_spank_* promoter present on pDR110 | This work |
|  | pGG29-30 | pMiniMAD2 derivate to create marker less in frame deletion of pLS20cat genes *29* and *30* | This work |
|  | pGG30 | pMiniMAD2 derivate to create in frame marker less deletion of pLS20cat gene *30* | This work |
|  | pCG35 | sf*GFP* gene cloned behind the P*_spank_* promoter present on pDR110 | This work |
|  | pCG36 | sf*GFP* gene cloned behind the P*_hyperspank_* promoter present on pDR111 | This work |
|  | pCG47 | sf*GFP* gene cloned behind the P*_xyl_* promoter present on pAX01 | This work |
|  | pCG52 | pMiniMAD2 derivate to create in frame marker less deletion of pLS20cat gene *29* | This work |
|  | pEST19 | pLS20cat genes *29* and *30* cloned behind the P*_spank_* promoter present on pDR110 | This work |
|  | pCGEST19 | pLS20cat genes *29* and *30* cloned behind the P*_hyperspank_* promoter present on pDR111 | This work |
|  | pCG106 | pLS20cat gene *29* cloned behind the P*_hyperspank_* promoter present on pDR111 | This work |
|  | pCG129 | pLS20cat gene *29* extended at its C-terminus with a region encoding a linker and a cMyc epitope (*ses-cMyc_LS20_*) was cloned behind the P*_hyperspank_* promoter present on pDR111 | This work |
| *, BGSC: Bacillus Genetic Stock Center, Department of Biochemistry, The Ohio State University, Columbus, OH, USA. (http://www.bgsc.org/ ) | | | |

**References**

Hartl, B., Wehrl, W., Wiegert, T., Homuth, G., and Schumann, W. (2001). Development of a new integration site within the Bacillus subtilis chromosome and construction of compatible expression cassettes. *J. Bacteriol* 183(8)**,** 2696-2699. doi: 10.1128/JB.183.8.2696-2699.2001 [doi].

Itaya, M., Sakaya, N., Matsunaga, S., Fujita, K., and Kaneko, S. (2006). Conjugational transfer kinetics of pLS20 between Bacillus subtilis in liquid medium. *Biosci. Biotechnol. Biochem* 70(3)**,** 740-742. doi: JST.JSTAGE/bbb/70.740 [pii].

Rudge, T.J., Federici, F., Steiner, P.J., Kan, A., and Haseloff, J. (2013). Cell polarity-driven instability generates self-organized, fractal patterning of cell layers. *ACS Synth. Biol* 2(12)**,** 705-714. doi: 10.1021/sb400030p [doi].

Steinmetz, M., and Richter, R. (1994). Plasmids designed to alter the antibiotic resistance expressed by insertion mutations in *Bacillus subtilis*, through in vivo recombination. *Gene* 142**,** 79-83.
