# Supplementary material for: Surface Exclusion Revisited: Function Related to Differential Expression of the Surface Exclusion System of Bacillus subtilis Plasmid pLS20
Source: Front Microbiol. 2019 Jul 10;10:1502. doi: 10.3389/fmicb.2019.01502 (PMC6635565; doi:10.3389/fmicb.2019.01502)
Supplement: Supplementary file 6 [file Table_3.docx]

| **Supplemental Table S3.** Oligonucleotides used | | |
| --- | --- | --- |
| **Name** | **Sequence (5’-3’)** | **Purpose** |
| oEST13 | tttt**GTCGAC**AAAAGGAGGTGAGTGTAAGTATGAGAAAG | Forward primer to amplify pLS20cat gene *29* (in combination with oEST17), or to amplify pLS20cat genes *29* and *30* (in combination with primer oEST14); *Sal*I restriction site extension |
| oEST14 | tttt**GCATGC**GCCCCTTTTTCTTTATTAAAAAACGTAAAGAACGG | Reverse primer to amplify pLS20cat gene *30* (in combination with primer oEST18), or to amplify pLS20cat genes *29* and *30* (in combination with primer oEST13); *Sph*I restriction site extension |
| oEST17 | tttt**GCATGC**TTATTATTACTGTCCTCTCTCATACACATCGTAGTTA | Reverse primer to amplify pLS20cat gene *29* in combination with primer oEST13; *Sph*I restriction site extension |
| oEST18 | cgat**GTCGAC**GAAGGAGGACAGTAAGCATTGGGTGAT | Forward primer to amplify pLS20cat gene *30* in combination with primer oEST14; *Sal*I restriction site extension |
| pDR111_L_sec | TTAAATGCAACCGTTTTTTCGGAAGG | Reverse primer to verify sequence of PCR fragments cloned in pDR110 or pDR111 |
| pDR111_U_sec | TGACTTTATCTACAAGGTGTGGC | Forward primer to verify sequence of PCR fragments cloned in pDR110 or pDR111 |
| D29_P1 | tttt**aagctt**GGCCGAGAAATACCTCTGG | Forward primer to amplify pLS20cat gene *29* “UP” region in combination with primer oCG28 or connectN29_C30_P5; *Hin*dIII restriction site extension |
| oCG26 | tatata**ggatcc**taagctagcactagTAAAGGAGGTGATCAAAATGGATT | Forward primer to amplify sfGFP gene in combination with primer oCG27 from the pKSsfGFP vector including upstream RBS. Giving PCR fragment of 900 bp. This primer also includes stop codons located upstream of the RBS to terminate possible translations of putative RBS sites located behind the P*_xyl_* promoter on pAX01 vector. Generated PCR fragment of primer set oCG26-oCG27 includes sfGFP gene with upstream stop codons followed by RBS, and also includes transcriptional terminator downstream of sfGFP gene. |
| oCG27 | attat**ggatcc**GTGTGACTCTAGTAGAGAGCGTTCACCG | Reverse primer to amplify *sfGFP* gene in combination with primer oCG27 from the pKSsfGFP vector including upstream RBS. This primer also includes stop codons located upstream of the RBS to terminate possible translations of putative RBS sites located behind the P*_xyl_* promoter on pAX01 vector. Generated PCR fragment of primer set oCG26-oCG27 includes *sf-gfp* gene with upstream stop codons followed by RBS, and also includes transcriptional terminator downstream of *sf-gfp* gene. |
| oCG28 | **cccattattaccactaccaacc**accagcagccaatttctttctcatacttacac | Reverse primer to amplify pLS20cat gene *29* “UP” region in combination with primer D29_P1. Contains a 5´-extension used in subsequent overlapping PCR to fuse the gene *29* “UP” region with the pLS20cat gene *29* “Down” region |
| oCG29 | gttggtagtggtaataatggg | Forward primer to amplify pLS20cat gene *29* “Down” region in combination with primer oCG30 |
| oCG30 | aaaa**gtcgac**ctcttctacaatcactatcgcgtt | Reverse primer to amplify pLS20cat gene *29* “Down” region in combination with primer oCG29; *Sal*I restriction site extension |
| oCG64 | agatccgcctccgcccTGTCCTCTCTCATACACATCGTAGTTATTACCTGTCCC | Reverse primer to amplify pLS20cat gene *29* in combination with primer oEST13, and template DNA pLS20cat. 5´ sequence (lower case) of primer oCG64 corresponds to sequence to fuse a “GGGGS” encoding linker to the last codon of gene *29.* |
| oCG67 | ttctgagatgagtttttgttcAGATCCGCCTCCGCCCTGTCCTCTCTCATACAC | Reverse primer to extend pLS20cat gene 29 containing C- terminal “GGGGS” encoding linker with part of cMyc epitope (corresponding to “EQKLISE” encoding region; indicated in lower case). Primer oCG67 was used in combination with primer oEST13, and template DNA corresponded to the PCR product oEST13-oCG64. |
| oCG68 | tttt**gcatgc**ttacagatcctcTTCTGAGATGAGTTTTTGTTCAGATCCGCCTCCGCCC | Reverse primer to extend further the pLS20cat gene 29 containing a C-terminal addition encoding a linker and part of cMyc epitope (“GGGGSEQKLISE”) to complete the cMyc tag. Primer oCG68 was used in combination with primer oEST13, and template DNA corresponded to the PCR product oEST13-oCG67. The extended region to complete the cMyc tag and corresponding to DNA encoding “EDL” followed by SphI cloning site are given in lower case |
| oPAX1up | gttgccgccgggcgttttttatgcagcaatg | Primer used to check, in combination with primer oPAX1dn, double cross-over integration of the P*_xyl_*-promoter containing cassette of pAX01 in the chromosomal *lacA* locus operon. This primer hybridizes to pAX01 sequence located downstream the N-terminal lacA region. PCR reaction using oPAX1up-opAX1dn gives 660 bp fragment in the case of a double cross-over event. |
| oPAX1dn | cagcataccggttgccgtcatctttattat | Primer used to check, in combination with primer oPAX1up, double cross-over integration of the P*_xyl_*-promoter containing cassette of pAX01 in the chromosomal *lacA* locus operon. This primer hybridizes to chromosomal sequence. PCR reaction using oPAX1up-opAX1dn gives 660 bp fragment in the case of a double cross-over event. |
| oPAX2up | cccccaagacttagcaagcgttttcattc | Primer used to check, in combination with primer oPAX2dn, double cross-over integration of the P*_xyl_*-promoter containing cassette of pAX01 in the chromosomal *lacA* locus operon. This primer hybridizes to chromosomal sequence. PCR reaction using oPAX2up-opAX2dn gives 620 bp fragment in the case of a double cross-over event. |
| opAX2dn | cccgggacgttcttgccattgctgcataa | Primer used to check, in combination with primer oPAX2up, double cross-over integration of the P*_xyl_*-promoter containing cassette of pAX01 in the chromosomal *lacA* locus operon. This primer hybridizes to pAX01 sequence located downstream the *xylR* gene. PCR reaction using oPAX2up-opAX2dn gives 620 bp fragment in the case of a double cross-over event. |
| opAX_seqDN | gttcacttaaatcaaagggggaaatg | Primer used to check insert cloned behind the P*_xyl_* promoter in pAX01 in combination with opAX_seqUP. A fragment of 229 bp will be generated using pAX01 as template. The size of the fragment will be enlarged with the size of the insert cloned behind the P*_xyl_*-promoter. |
| opAX_seqUP | caggtggcacttttcggggaaatgtg | Primer used to check insert cloned behind the P*_xyl_* promoter in pAX01 in combination with opAX_seqDN. A fragment of 229 bp will be generated using pAX01 as template. The size of the fragment will be enlarged with the size of the insert cloned behind the P*_xyl_*-promoter. |
| D29_P1 | ttttaagcttGGCCGAGAAATACCTCTGG | Forward primer to amplify pLS20cat gene *29* “UP” region in combination with primer oCG28 and by the other hand with connectN29_C30_P5 ; *Hin*dIII restriction site extension |
| oCG28 | **CCCATTATTACCACTACCAAC**accagcagccaatttctttctcatacttacac | Reverse primer to amplify pLS20cat gene *29* “UP” region in combination with primer D29_P1. Contains a 5´-extension used in subsequent overlapping PCR to fuse the gene *29* “UP” region with the pLS20cat gene *29* “Down” region |
| oCG29 | gttggtagtggtaataatggg | Forward primer to amplify pLS20cat gene *29* “Down” region in combination with primer oCG30 |
| oGG30 | aaaa**gtcgac**ctcttctacaatcactatcgcgtt | Reverse primer to amplify pLS20cat gene *29* “Down” region in combination with primer oCG29; *Sal*I restriction site extension |
| connectN29_C30_P5 | **ctgaggtgtcaaactctactaa**ctttctcatacttacactcaacacc | Reverse primer to amplify pLS20cat gene 29 “UP”region in combination with primer d29_1. Contains 5´ extension used in subsequent overlapping PCR to fuse the gene 29 “UP” region with the pLS20cat gene 30 “Down” region |
| D29_p6 | ttagtagagtttgacacctcag | Forward primer to amplify pLS20cat gene *30* “Down” region in combination with primer D29_P7 |
| D29_30_p7 | aaaa**gtcgac**aattgtggtacttcaattgtcggcgggt | Reverse primer to amplify pLS20cat gene 30 “Down” region in combination with primer D29_P6; *Sal*I restriction site extension |
| 29_UpHindIII | tttt**AAGCTT**CTGAATAAAGTGAGGTTGATCAGG | Forward primer to amplify 382 bp region upstream of pLS20cat gene *29* in combination with primer 29_DnHindIII; *Hin*dIII restriction site extension |
| 29_DnHinIII | tttt**AAGCTT**CCATTCACAATTTTACTATGGTTTGAG | Reverse primer to amplify 382 bp region upstream of pLS20cat gene *29* in combination with primer 29_UpHindIII; *Hin*dIII restriction site extension |
| D30_p8 | aaaa**aagctt**gaaacgctaaaaaagagaattgaagatc | Forward primer to amplify pLS20cat gene *30* “UP” region in combination with primer connectN29_C30_P9; *Hin*dIII restriction site extension |
| Connect_C29_C30_P9 | **ctgaggtgtcaaactctactaa**atcacccaatgcttactgtcctctctc | Reverse primer to amplify pLS20cat gene *30* “UP” region in combination with primer D29_P6. Contains a 5´-extension used in subsequent overlapping PCR to fuse the gene *30* “UP” region with the pLS20cat gene *30* “Down” region |
| oCG11 | attat**gtcgac**gtgtgactctagtagagagcgttcaccg | Reverse primer to amplify sf-*gfp* gene (873bp) of pKSsfGFP vector in combination with primer oCG12; *Sal*I restriction site extension |
| oCG12 | tttt**aagctt**gctagcactagtaaaggaggtgatcaaaatggatt | Forward primer to amplify sf-*gfp* gene (873bp) of pKSsfGFP vector in combination with primer oCG11; *Hin*dIII restriction site extension |
| oCG14 | ACATTTCTTCTTGGCGCGAGTGACGGTT | Forward primer to verify deletion of gene *29* in pLS20cat |
| oCG15 | GGTGTCAAACTCTACTAAGTATAA | Reverse primer to verify deletion of gene *29* in pLS20cat |
| oCG16 | ACGGTTTTCACCGGTCGTAGACGGCAAGTT | Forward primer to verify deletion of genes *29-30* in pLS20cat |
| oCG17 | TTACTGTGCGATCCCAGCTCTCACTGCT | Reverse primer to verify deletion of genes *29-30* in pLS20cat |
| oCG18 | CAGGTAGCTCGTCCAATTCAAGCA | Forward primer to verify deletion of gene *30* in pLS20cat |
| oCG19 | CTATTCGATCATTTATCCTCTTCTA | Reverse primer to verify deletion of gene *30* in pLS20cat |
| oSeqpJS104_Dn | CGCGATTTCCAATGAGGTTAAGAGTA | Forward primer to verify sequence of PCR fragments cloned in pKSsfGFP |
| oSeqpKSGFP_Dn | TTCCGGCATGGCGGACTTGAAGAAGTC | Reverse primer to generate deletion of constitutive promoter in pJS104 to construct pKSsfGFP. Reverse primer to verify sequence of PCR fragments cloned in pKSsfGFP |
| oPKS45 | TTTGAATTCGCTAGCACTAGTAAAGGAGGTGATCAAAATGGATTCAATAGAAAAGGTAAG | Froward primer to generate deletion of constitutive promoter in pJS104 to construct pKSsfGFP. |
| Prom28UP_Hind | CCCCAAGCTTTATACCACCTCGCAAAATAAACC | Primer to amplify the 583 bp region encompassing pLS20cat promoter P*_c_* in combination with Prom28Dn_Bam |
| Prom28Dn_Bam | CGCGGATCCCCTAAATTTTCAATCAGTGTAAAG | Primer to amplify the 583 bp region encompassing pLS20cat promoter P*_c_* in combination with Prom28UP_Hind |
| pMiniMAD2seqDn | ttgggaccgcaatgggttgaatta | Reverse primer to verify sequence of PCR fragments cloned in pMiniMAD2 |
| pMiniMAD2seqUP | TATGTTGTGTGGAATTGTGAGCGGA | Forward primer to verify sequence of PCR fragments cloned in pMiniMAD2 |
| M13RevExt | Cacaggaaacagctatgaccatg | Reverse primer to verify sequence of PCR fragments cloned in pMiniMAD2 |
| M13_Fw-21_ext | Acgttgtaaaacgacggccagtg | Forward primer to verify sequence of PCR fragments cloned in pMiniMAD2 |
| 5´-overhang sequences to optimize digestion of restriction enzymes are indicated in lower case. Restriction sites are underlined and shown in red. 5´-overhang sequences that function in overlapping PCR reactions are shown in green | | |
