# Supplementary material for: Surface Exclusion Revisited: Function Related to Differential Expression of the Surface Exclusion System of Bacillus subtilis Plasmid pLS20
Source: Front Microbiol. 2019 Jul 10;10:1502. doi: 10.3389/fmicb.2019.01502 (PMC6635565; doi:10.3389/fmicb.2019.01502)
Supplement: Supplementary file 7 [file Table_4.docx]

|  | **Supplemental Table S4.** homologs of Ses_LS20_  and Ses_576_ | | | | | |
| --- | --- | --- | --- | --- | --- | --- |
|  | **Accession Nº** | **Phylum** | **Organism** | **N-ter TMD^α^** | **conj operon (gene Nº operon)*** | **Plasmid^#^** |
|  | [WP_013603190.1](https://www.ncbi.nlm.nih.gov/protein/503368529?report=genbank&log$=prottop&blast_rank=1&RID=CDBNJJ41016) | Firmicutes | *Bacillus subtilis* | +^β^ | + (2) | pLS20 |
|  | [WP_072176319.1](https://www.ncbi.nlm.nih.gov/protein/1118255384?report=genbank&log$=prottop&blast_rank=2&RID=CDBNJJ41016) | Firmicutes | *Bacillus subtilis* | + | + (2) | + |
|  | WP_095431340.1 | Firmicutes | *Bacillus sp. X2* | + | + (2) | + |
|  | [WP_061671007.1](https://www.ncbi.nlm.nih.gov/protein/1005579256?report=genbank&log$=prottop&blast_rank=3&RID=CDBNJJ41016) | Firmicutes | *Bacillus atrophaeus* | + | + (2) | + |
|  | [WP_073982108.1](https://www.ncbi.nlm.nih.gov/protein/1122245449?report=genbank&log$=prottop&blast_rank=4&RID=CDBNJJ41016) | Firmicutes | *Bacillus amyloliquefaciens* | + | + (2) | + |
|  | KDN91413.1^§^ | Firmicutes | *Bacillus amyloliquefaciens* | + | + (2) | + |
|  | [WP_073461406.1](https://www.ncbi.nlm.nih.gov/protein/1120947210?report=genbank&log$=prottop&blast_rank=5&RID=CDBNJJ41016) | Firmicutes | *Bacillus licheniformis* | + | + (2) | + |
|  | [WP_061578161.1](https://www.ncbi.nlm.nih.gov/protein/1005283035?report=genbank&log$=prottop&blast_rank=6&RID=CDBNJJ41016) | Firmicutes | *Bacillus licheniformis* | + | + (2) | + |
|  | [WP_061573930.1](https://www.ncbi.nlm.nih.gov/protein/1005278266?report=genbank&log$=prottop&blast_rank=7&RID=CDBNJJ41016) | Firmicutes | *Bacillus amyloliquefaciens* | + | ? | - |
|  | PAK32451.1 | Firmicutes | *Bacillus safensis* | + | + (2) | + |
|  | [WP_047947281.1](https://www.ncbi.nlm.nih.gov/protein/844815997?report=genbank&log$=prottop&blast_rank=8&RID=CDBNJJ41016) | Firmicutes | *Bacillus altitudinis* | + | + (2) | + |
|  | WP_039076349.1 | Firmicutes | *Bacillus MSP13* | + | ? | + |
|  | KIL24029.1 | Firmicutes | *Bacillus pumilus* | + | + (7) | + |
|  | [WP_075623812.1](https://www.ncbi.nlm.nih.gov/protein/1130456993?report=genbank&log$=prottop&blast_rank=19&RID=CDBPK86B016) | Firmicutes | *Bacillus safensis* | + | ? | unnamed plasmid 1 |
|  | WP_101356204.1 | Firmicutes | *Bacillus camelliae* | + | +(3) | + |
|  | WP_105928603.1 | Firmicutes | *Bacillus sp.* LLTC93 | + | +(2) | + |
|  | WP_106031812.1 | Firmicutes | *Bacillus sp.* NMCC4 | + | +(2) | + |
|  | WP_107982029.1 | Firmicutes | *Bacillus sporothermodurans* | + | +(3) | + |
|  | WP_061140925.1 | Firmicutes | *Bacillus simplex* | ± | - | - |
|  | WP_105322206.1 | Firmicutes | *Bacillus velezensis* CGMCC | + | +(2) | unnamed plasmid |
|  | AGK54042.1 | Firmicutes | *Bacillus sp.* 1NLA3E | + | - | - |
|  | WP_077721637.1 | Firmicutes | *Bacillus velezensis* | + | - | unnamed plasmid |
|  | [WP_058831713.1](https://www.ncbi.nlm.nih.gov/protein/970574554?report=genbank&log$=prottop&blast_rank=11&RID=CDBNJJ41016) | Firmicutes | *Listeria monocytogenes* | + | + (?) | ? |
|  | [WP_003759254.1](https://www.ncbi.nlm.nih.gov/protein/489855591?report=genbank&log$=prottop&blast_rank=12&RID=CDBNJJ41016) | Firmicutes | *Listeria grayi* | + | + (6), | + (pLGUG1) |
|  | [WP_031659815.1](https://www.ncbi.nlm.nih.gov/protein/685924855?report=genbank&log$=prottop&blast_rank=14&RID=CDBNJJ41016) | Firmicutes | *Listeria monocytogenes* | + | + (?) | ? |
|  | WP_012952139.1 | Firmicutes | *Listeria monocytogenes* | + | + (4) | + (pLM5578) |
|  | WP_021430680.1 | Firmicutes | *Paraclostridium bifermentans* | + | - | ? |
|  | [WP_057553342.1](https://www.ncbi.nlm.nih.gov/protein/950968280?report=genbank&log$=prottop&blast_rank=17&RID=CDBPK86B016) | Firmicutes | *Paeniclostridium sordelli* | + | + (1) | + |
|  | SCJ52122.1 | Firmicutes | *Clostridium sp.* | + | + (?) | + |
|  | [WP_021434480.1](https://www.ncbi.nlm.nih.gov/protein/545061392?report=genbank&log$=prottop&blast_rank=20&RID=CDBPK86B016) | Firmicus | *Paraclostridium bifermentans* | + | + (1) | + |
|  | α, predicted N-terminal transmembrane spanning domain. *, conjugation operon, symbol “+” indicates that the identified gene is located in a (putative) conjugation operon; the number in brackets indicates the position of the identified gene within the operon. When the identified gene is located at the beginning of a contig, or the contig is only composed of a single or few genes, the position of the gene within the operon could not be determined (indicated with “’?”). #, symbol “+” indicates that the gene is present on a plasmid; when possible the name of the plasmid is given in brackets. ?, indicates that there is insufficient data in the database to discern whether the gene is located on a plasmid or not. ^§^, the sequence deposited in the database probably contains a one-bp deletion (C) at codon 30. β, “+” and “±” (only in the case of KDN91413.1) indicates that a transmembrane domain is predicted with more than 95% or 60% confidence, respectively. | | | | | |
