# Supplementary material for: Surface Exclusion Revisited: Function Related to Differential Expression of the Surface Exclusion System of Bacillus subtilis Plasmid pLS20
Source: Front Microbiol. 2019 Jul 10;10:1502. doi: 10.3389/fmicb.2019.01502 (PMC6635565; doi:10.3389/fmicb.2019.01502)
Supplement: Supplementary file 8 [file Table_5.docx]

| **Supplemental Table S5**. Synteny of genes *29* - *30* in pLS20cat is not conserved in most other contigs containing a Ses_pLS20_ homolog | | | | |
| --- | --- | --- | --- | --- |
| **Organism** | **Ses_pLS20_ (homolog)^1^** | **Accession Nº protein downstream gene^2^** | **Nº residues^3^** | **Identity (%)of deduced protein with p30 of pLS20cat** |
| *Bacillus subtilis* | WP_072176319.1 | WP_072176328.1 | 767 | 3.1 |
| *Bacillus sp.* X2 | WP_095431340.1 | WP_095431339.1 | 774 | 3.1 |
| *Bacillus atrophaeus* | WP_061671007.1 | WP_061671008.1 | 93 | 30.4 |
| *Bacillus amyloliquefaciens* | WP_073982108.1 | WP_073982107.1 | 94 | 17.5 |
| *Bacillus amyloliquefaciens* | KDN91413 | KDN91412.1 | 94 | 17.5 |
| *Bacillus licheniformis* | WP_073461406.1 | WP_073461455.1 | 765 | 2.7 |
| *Bacillus licheniformis* | WP_061578161.1 | WP_081112690.1 | 765 | 3.2 |
| *Bacillus amyloliquefaciens* | WP_061573930.1* | - | - | - |
| *Bacillus safensis* | PAK32451.1 | PAK32452.1 | 61 | 9.8 |
| *Bacillus altitudinis* | WP_047947281.1 | WP_047947282.1 | 61 | 9.2 |
| *Bacillus sp.* MSP13 | WP_039076349.1 | WP_082022846.1 | 766 | 2.4 |
| *Bacillus pumilus* | KIL24029.1 | KIL24028.1 | 715 | 4.1 |
| *Bacillus safensis* | WP_075623812.1 | WP_075623813.1 | 770 | 3.0 |
| *Bacillus camelliae* | WP_101356204.1 | WP_101356203.1 | 851 | 2.1 |
| *Bacillus sp.* LLTC93 | WP_105928603.1 | WP_105928602.1 | 103 | 2.1 |
| *Bacillus sp.* NMCC4 | WP_106031812.1 | WP_106031811.1 | 103 | 7.5 |
| *Bacillus sporothermodurans* | WP_107982029.1 | WP_107982028.1 | 771 | 3.5 |
| *Bacillus simplex* | WP_061140925.1 | [WP_061140926.1](https://www.ncbi.nlm.nih.gov/protein/1000884926) | 173 | 15.0 |
| *Bacillus velezensis* | WP_105322206.1 | WP_073982107.1 | 94 | 17.5 |
| *Bacillus sp.* 1NLA3E | [WP_051120151.1](https://www.ncbi.nlm.nih.gov/protein/916449990?report=genbank&log$=prottop&blast_rank=15&RID=CDBNJJ41016) | WP_041580489.1 | 125 | 11.6 |
| *Bacillus velezensis* | WP_077721637.1 | WP_077721679.1 | 760 | 3.3 |
| *Listeria monocytogenes* | WP_058831713.1 | WP_058831714.1 | 75 | 8.8 |
| *Listeria grayi* | WP_003759254.1 | WP_003759252.1 | 105 | 7.1 |
| *Listeria monocytogenes* | WP_031659815.1 | WP_031659817.1 | 119 | 9.7 |
| *Listeria monocytogenes* | WP_012952139.1 | WP_012952140.1 | 128 | 7.1 |
| *Paraclostridium bifermentans* | WP_021434480.1 | WP_021434431.1 | 783 | 2.8 |
| *Paeniclostridium sordellii* | WP_057553342.1 | WP_057553341.1 | 782 | 3.0 |
| *Clostridium sp.* | SCJ52122.1 | SCJ52104.1 | 782 | 3.8 |
| *Paraclostridium bifermentans* | WP_021434480.1 | WP_021434431.1 | 783 | 2.8 |
| ^1^, Accession number of the Ses_pLS20_ homolog (see Table S4); ^2^, Accession number of the protein encoded by the downstream located gene; ^3^ Number of residues of the protein encoded by the gene located downstream of the *ses_pLS20_* homolog; ^4^, identity (%) of the protein encoded by the downstream located gene with protein p30 encoded by pLS20cat.  *, WP_061573930.1 is encoded by the last gene of the deposited contig. | | | | |
